# Experimental trichuriasis: Changes in the immune response and bacterial translocation during acute phase development illustrated with 3D model animation

**DOI:** 10.1371/journal.pntd.0012841

**Published:** 2025-02-03

**Authors:** Dayane Alvarinho de Oliveira, Renato Oliveira, Brunna Vianna Braga, Lorian Cobra Straker, Luciana Silva Rodrigues, Lilian Lacerda Bueno, Ricardo Toshio Fujiwara, Eduardo José Lopes-Torres

**Affiliations:** 1 Laboratório de Helmintologia Romero Lascasas Porto, Departamento de Microbiologia, Imunologia e Parasitologia, Faculdade de Ciências Médicas, Universidade do Estado do Rio de Janeiro, Rio de Janeiro, Brazil; 2 Laboratório Multiusuário de Parasitologia, Departamento de Microbiologia, Imunologia e Parasitologia, Faculdade de Ciências Médicas, Universidade do Estado do Rio de Janeiro, Rio de Janeiro, Brazil; 3 Laboratório de Evolução e Biologia Integrativa, Departamento de Biologia, Faculdade de Filosofia, Ciências e Letras de Ribeirão Preto, Universidade de São Paulo, São Paulo, Brazil; 4 Centro Nacional de Biologia Estrutural e Bioimagem, Universidade Federal do Rio de Janeiro, Rio de Janeiro, Brazil; 5 The Francis Crick Institute, London, England, United Kingdom; 6 Laboratório de Imunopatologia, Departamento de Patologia e Laboratórios, Faculdade de Ciências Médicas, Universidade do Estado do Rio de Janeiro, Rio de Janeiro, Brazil; 7 Departamento de Parasitologia, Instituto de Ciências Biológicas, Universidade Federal de Minas Gerais, Belo Horizonte, Brazil; Consejo Nacional de Investigaciones Cientificas y Tecnicas, Fundación Mundo Sano, ARGENTINA

## Abstract

Trichuriasis, a well-known type of soil-transmitted helminthiasis, is a neglected gastrointestinal nematode disease predominantly affecting children in tropical regions and is caused by *Trichuris trichiura*. The potential zoonotic transmission of this disease is indicated by its presence in nonhuman primates. Chronic infection leads to mucosal damage, bacterial translocation, and intense inflammatory infiltration; however, the progression of these processes remains poorly understood. This study tracks the acute phase of experimental trichuriasis, providing detailed insights into nematode tissue migration stages, inflammatory infiltration, cytokine production, and 2D/3D imaging of the bacterial translocation process. We showed a mixed immune response (Th1, Th2, and Th17) initiated by larval-induced lesions in the intestine tissue and modulated by L4 larvae and adult worms in the cecum, with systemic changes observed in the mesenteric lymph nodes, peritoneal macrophages, and spleen. Despite the disruption of the intestinal mucosa within the first 10 days post-infection (d.p.i.), bacterial invasion becomes evident only after the development of the nematode into the L3 larval stage (17 d.p.i.), intensifying with lesions caused by the L4 larvae (22 d.p.i.) and adult worms (35 d.p.i.). Our multidimensional approach, which incorporates microscopy tools, micro-CT, physiological evaluations, tissue/organ assessments, and immunological parameters, demonstrates the ability of larvae to breach the intestinal mucosa, further indicating the timing of extensive bacterial infiltration. Additionally, a 3D animation illustrates how adult worm attachment mechanisms may facilitate bacterial translocation. This study provides significant insights into the immunological and pathological mechanisms of trichuriasis progression, highlighting the complex interplay among host immune responses, the gut microbiome, and parasite survival strategies, all of which are crucial aspects for future therapeutic development.

## Author summary

Soil-transmitted helminthiases, such as trichuriasis, predominantly affect children in tropical regions with inadequate sanitation. These infections, caused by whipworms, damage the intestinal tissue, disrupt the balance of microbiota, cause bacterial translocation, and trigger immune responses with significant health consequences. Our study focuses on understanding the progression of the infection – from the ingestion of infective eggs and the initiation of mucosal damages (acute phase) to the establishment of chronic infection - particularly highlighting how the parasite facilitates bacterial invasion through damaged tissue. Using advanced techniques that integrate immunology experiments, 3D imaging, and animation, we explore the early stages of infection and demonstrate how the parasite’s movements and attachment mechanisms enable bacteria to cross the intestinal barrier. Additionally, we emphasize the potential of 3D models in enhancing the understanding of host-parasite interactions in large-volume biological sample. These models allow students and educators to visualize complex biological processes in a more engaging way and provide inclusive learning opportunities for visually impaired individuals, fostering greater accessibility in science education. By shedding light on the intricate interactions among neglected parasites, bacteria, and the host immune system, our research advances knowledge in parasitology and suggests new avenues for developing strategies to combat these often-neglected diseases.

## Introduction

Trichuriasis, a well-known type of helminthiasis transmitted from soil, is a neglected tropical disease caused by the nematode *Trichuris trichiura*, affecting approximately 500 million people worldwide [1] in underserved communities, mainly in southern and southwestern China, Southeast Asia, southern India, sub-Saharan Africa, and Central and South America, where basic sanitation and clean water are inadequate or absent [1].

The symptomatology of this infection is nonspecific, with clinical presentations including nausea, fatigue, flatulence, abdominal distension and pain. However, the severity of symptoms may increase depending on the parasitic load and the host’s immunological characteristics, which can include iron deficiency anemia, loss of appetite, weight loss, imbalance in the intestinal microbiota, mucoid diarrhea, and intestinal bleeding, and in severe cases cause rectal prolapse [2], the impacts of which are associated with tissue damage caused by the nematode and bacterial translocation [3]. Moreover, individuals with preexisting comorbidities, such as autoimmune rheumatic diseases and immunosuppressed patients, face a greater risk of experiencing severe manifestations of intestinal parasitic infections, potentially leading to fatal outcomes [4,5]. Helminthiasis induces damage to the host’s body at specific parasite niches and in other organs distant from the location of the parasites [6,7]. Furthermore, helminth infections may compromise the host’s ability to develop protective immune responses against vaccines [8] and bacterial infection [9]. Additionally, nonhuman primates infected by *T. trichiura*, as reported in African green monkeys (AGM, *Chlorocebus sabaeus*), indicate a potential zoonotic risk, opening new challenges in epidemiological vigilance [10].

The life cycle of *Trichuris* spp. begins when a mammalian host ingests embryonated eggs containing larvae at the L1 stage. In experimental murine models, these eggs become sensitized due to the action of intestinal microbiota bacteria, particularly *Escherichia coli* with type I fimbriae and *Staphylococcus aureus*, which adhere to the polar plugs and induce disintegration, thus enabling larval hatching [11,12]. Approximately 90 min after ingestion, the embryonated eggs reach the cecum, and the L1 larvae hatch and degrade the mucosal layer to penetrate the intestinal epithelial cells at the base of the Lieberkühn crypts [13]. The larvae migrate through the tissue, forming an initial structure of the syncytial epithelial tunnel [14,15]. After 24 h, the L1 larvae become intracellular [13]. Approximately 17 days later, L2 larvae molt into L3, thereafter into L4 at 22 days (post-infection), and by 32 days, adult worms are sexually mature, with bacillary band structures developing [7,15].

In the chronic stage, 35 days of infection, the adult worm will be partially inserted into the tissue, where the anterior region remains inserted in the intestinal epithelium and the posterior region protrudes into the lumen, causing tissue rupture and points of contact for the intestinal microbiota [14]. However, little is known about when bacterial translocation begins during trichuriasis infection and what its true bacterial impact is, even after elimination of the worm. Furthermore, the bacillary glands, present in the anterior region, are capable of absorbing nutrients [14,16] and secreting immunomodulatory products through its 50,000 pores [3,17]. Therefore, trichuriasis infection evolution involves larval, egg and adult stages, each with antigenic and immunomodulatory properties [18–20].

The immune response generated against *T. muris* during the early days of infection determines susceptibility or resistance to infection [7,15]. In the chronic phase with a high parasitic load, murine trichuriasis involves a mixed Th1/Th2/Th17 immune response; however, in the cecum, only IFN-γ levels are increased, demonstrating the immunomodulatory capacity of the parasite [3]. Although the parasite releases substances that prevent excessive tissue damage, studies with C57BL/6 APC min/+ mice have shown that trichuriasis can induce neoplastic alterations in the intestine, increasing the proliferation and size of carcinogenic cells in animals with tumor predisposition genes [21,22].

In this study, our goal was to comprehensively investigate the progression of trichuriasis in C57BL/6 mice, with a particular focus on the impact of a low parasitic dose. We present the association between the parasite’s attachment, which facilitates bacterial translocation through tissue damages, and the new observation that bacteria adhered to the nematode cuticle could be internalized into the submucosa, driven by the parasite’s movement. Our research spanned from the acute phase (at 90 minutes, 10 days, 17 days, and 22 days post-infection) to the chronic phase (at 35 days post-infection) and employed a multidimensional approach, incorporating histopathology, physiological evaluations, assessments of tissue/organ alterations, and immunological parameters. Moreover, to explore the interaction of the parasite and the ability of the larvae to breach the intestinal mucosa, we used advanced ultrastructural analyses, including electron microscopy, fluorescence in hybridization (FISH), and micro-CT, in highly infected Swiss mice to understand the timing of extensive bacterial translocation into the intestinal submucosa. Additionally, using 3D animation, we illustrate how the attachment mechanism of adult worms may contribute to bacterial translocation. These findings provide new insights into the complex dynamics of the relationship between whipworms and the microbiota, thereby enhancing our understanding of this neglected disease.

## Materials and methods

### Ethics statement

The parasite’s life cycle was maintained in the animal facility of the Department of Parasitology, UERJ. The animals received humanized care in compliance with Brazilian federal legislation (Law 11,794/2008, regulated by Decree 6,899/2009), following the guidelines outlined in the “Guide for the Care and Use of Laboratory Animals” by the US National Academy of Science and the “Australian Code of Practice for the Care and Use of Animals for Scientific Purposes” [23]. All procedures involving animals were thoroughly reviewed and approved by the ethics committee of the Instituto de Biologia Roberto Alcântara Gomes of UERJ and were conducted in accordance with CEUA 059/2018.

### Experimental infections

Animal experiments were conducted using 75 C57BL/6 mice (isogenic strain) and 10 Swiss specific pathogen-free mice. All mice were male and four weeks old. These mice were obtained from the animal facility of ICTB/Fiocruz Manguinhos and maintained in the Department of Parasitology facility at UERJ. The C57BL/6 animals were organized into ten groups in two different experimental infection groups: five control groups (noninfected) and five infected groups with 50 embryonated eggs of *Trichuris muris* (low-dose) corresponding to different infection time points (90 minutes, 10, 17, 22, and 35 days post-infection (d.p.i.)). The *T. muris* eggs used for experimental infection were obtained from the Edinburgh strain. In previous infections, the worms were isolated and maintained in RPMI 1,640 culture medium (Cultilab) supplemented with gentamicin (10 mg/ml) and amphotericin (2.5 μg/ml) in a CO2 incubator at 27°C for 1 hour. Subsequently, the worms were washed in sterile 0.9% saline and transferred to RPMI 1,640 medium (Cultilab) without supplements, where they were incubated in a CO_2_ at 27°C for 24 hours. After this incubation period, the medium containing the eggs was centrifuged at 500 × g for 5 minutes. The eggs were then washed twice with dechlorinated/sterile water, and incubated at 28 ± 2°C for 45 days. The percentage of embryonated eggs was subsequently estimated using light microscopy. The noninfected groups received only sterile water at the same volume of 0.15 mL. For specific microscopy experiments (SEM and FISH) and micro-CT, 10 male Swiss mice were infected with a high dose of *T*. *muris* following the protocol described by [3], due to the greater quantity of parasites in the tissue and better visualization for microscopy techniques. The temperature of the C57BL/6 animals was measured immediately before euthanasia via an infrared thermometer (Central Brasil model CB-88ª, range −50 to 420 °C). Measurements were taken toward the animal’s abdomen, maintaining an approximate distance of 132 mm, as per the manufacturer’s instructions and following the protocol of [24].

### Ultrastructure of the intestine infected with *Trichuris muris*

For scanning electron microscopy (SEM), during necropsy, Swiss cecum samples were removed, washed in sterile 0.9% saline, subsequently fixed in 4% formalin and processed via a conventional SEM protocol [17]. The samples were dehydrated in a graded series of ethanol (30% to absolute), fractured according to [25], critical point dried with liquid CO_2_, mounted on stubs, coated with gold (20–25 nm), and examined via a scanning electron microscope AURIGA Compact Zeiss (Zeiss, Germany) at the Urogenital Unit of UERJ.

### Morphometric and morphological experiments

Large intestine fragments from the cecum region of C57BL/6 mice were fixed in 8% formaldehyde at pH 7.4 for 24 h and transferred to 4% formalin. The tissue was then dehydrated for 15 minutes in a gradual series of ethanol (30% to absolute), diaphanized with xylene (Merck) twice for 15 minutes each, and embedded in paraffin (Sigma-Aldrich), two step of 30 minutes. Five-micron-thick tissue sections were stained with hematoxylin/eosin (Sigma‒Aldrich), PAS (Sigma‒Aldrich), and Giemsa (Merck). Morphometric and morphological analyses were conducted via an Olympus BX 53 microscope equipped with an Olympus SC100 digital camera and CellSensEntry software version 1.18, as well as Bel View version 6.2.3.0 (Bel Engineering, Monza Italy). The three layers of the cecum were measured in five different random areas from five animals in each group (noninfected and infected) at each time point of infection (90 min and 10, 17, 22, and 35 days). For mucosal and submucosal morphological analyses, cells were quantified and identified in three randomly selected areas of 50 µm^2^ in ten animals from each group. For enterocyte and goblet cell quantification, three crypts of Lieberkühn were randomly selected from four animals from each group (uninfected and infected) for each infection and analysis.

### Weighing of the mouse body, spleen and cecum (g)

Live C57BL/6 animals were placed in a 500 mL clear plastic container and weighed on Days 0, 10, 17, 22, and 35 post-infection, as well as immediately before euthanasia. During necropsy, the spleen and cecum were removed and weighed. The spleen was subsequently photographed to measure its length using ImageJ 1.53K software. The ratio of the spleen weight (g) to the mouse body weight was calculated and expressed as a percentage. The cecum was weighed with and without feces in 90 × 15 mm Petri dishes and washed with 0.9% saline solution. The volume of feces in the cecum was determined by calculating the difference between the cecum when full and when feces were not present. Following imaging, the spleens were fixed by immersion in 4% phosphate-buffered formalin.

### Hematological analysis

To conduct specific and differential leukocyte counts, blood distention was performed. Approximately 5μL of peripheral blood obtained from the tail of C57BL/6 animals was added onto a clean and degreased glass slide and distended at a 45° angle. After drying at room temperature, the cells were fixed and stained via a Panoptic Staining Kit (Laborclin). The slides were then examined under a light microscope with immersion, utilizing the Olympus Standard microscope model CX 21.

### Peritoneal murine macrophage isolation and *in vitro* experiments

Peritoneal macrophages were obtained by washing the peritoneal cavity of C57BL/6 mice with 5 mL of Roswell Park Memorial Institute (RPMI) medium containing gentamicin (10 mg/mL) and amphotericin (2.5 μg/mL), which had been previously refrigerated at 4 °C. The total number of viable cells from the mice, whether or notthey were infected with *T. muris*, was determined via Trypan blue (Sigma‒Aldrich) in a Neubauer chamber. The macrophages were then plated at a concentration of 2 × 10^6^ cells and then allowed to adhere to the plate for one hour in a CO_2_ incubator at 37 °C. After adherence, the supernatant was discarded, and RPMI + FBS (10%) + LPS (3 μg/mL) medium was added in duplicate, with the other duplicate receiving only RPMI + FBS (10%) per animal. The cells were then incubated for 24 hours in a CO_2_ incubator at 37 °C, and the supernatant was aliquoted and immediately frozen at −80 °C until cytokine measurement. Nitric oxide (NO) production was assessed by measuring the concentration of nitrite (NO^-2^) in the supernatant using the Griess colorimetric method. In summary, peritoneal macrophages from noninfected or infected mice were stimulated with 3 μg/mL LPS for 24 hours, whereas the other macrophages remained unstimulated. The amount of NO^-2^in the supernatants was then measured. The absorbance was measured spectrophotometrically at 570 nm, and values were obtained via calculation via the standard curve of sodium nitrite (NaNO_2_).

### Cytokine measurements in immune organs and cell culture supernatants

Lymph nodes (LNs) and large intestine fragments were collected and homogenized for cytokine analysis. Each 1 mg of LN was homogenized in 100 μl of PBS. For large intestine fragments, the same weight (1 mg) of 50 μl of PBS was used. The organs and tissues were immersed in ice-cold PBS, macerated, and passed through a 70-μm-mesh-size cell strainer (BD). After homogenization, the samples were centrifuged at 4 °C and 500 × g for 15 min, and the supernatant was removed and immediately frozen at −80 °C until use. To quantify cytokines, we measured five different infection time points: 90 min and 10, 17, 22, and 35 days post-infection (d.p.i.). Cytokine levels (IL-2, IL-4, IL-6, IFN-γ, TNF, IL-17A, and IL-10) in LNs and large intestine fragment supernatants were determined using commercial kits (cytometric bead array [CBA] mouse Th1/Th2/Th17; BD Pharmingen). Cytokine levels (IL-6, IL-10, MCP-1, IFN-γ, TNF, and IL-12p70) in the peritoneal macrophage supernatant were determined via a commercial kit (cytometric bead array mouse CBA kit for inflammatory cytokines; BD Pharmingen). The supernatants were incubated with cytokine-specific antibody-coated microspheres and a phycoerythrin (PE)-conjugated detection antibody at room temperature for 2 h in the dark. A standard curve was prepared by incubating the microspheres at different concentrations (20 to 5,000 pg/ml). After incubation, 500 μl of wash solution was added to each tube and centrifuged at 200 × g for 5 min. The supernatant was discarded, and the pellet was resuspended in 300 μl of wash solution. Data were acquired on a FACSCanto II flow cytometer (BD Bioscience, USA) and analyzed via FCAP Array software (BD Bioscience). The cytokine detection limits (pg/mL) were as follows: IL-2, 0.1; IL-4, 0.03; IL-6, 1.4; IFN-γ, 0.5; TNF, 0.9; IL-17A, 0.8; and IL-10, 16.8. Cytokine concentrations were determined on the basis of the manufacturer´s recommendation.

### Bacterial identification

The fluorescence *in situ* hybridization (FISH) experiments were conducted using Swiss cecum fragments fixed in 4% formalin for 48 hours. Following fixation, the tissue was incubated at 4 °C in 10% and 30% sucrose for 24 hours, embedded in OCT gel (Tissue-Tek) and frozen in liquid nitrogen. Thin sections (5 µm) were obtained and mounted on slides coated with poly-L-lysine (Sigma–Aldrich) via a cryostat (Leica CM1850). FISH was performed on each slide according to previously described conditions and buffers [26], with hybridization buffer containing 30% formamide and probes targeting eubacteria EUB338; EUB338 II; EUB338 III, and NONEUB (control) from Thermo Fisher Scientific. The slides were subsequently stained with DAPI at a concentration of 0.1 µg/ml for 10 minutes, coated with N-propyl gallate (Sigma–Aldrich). The analyses performed on Zeiss fluorescence microscopes equipped with AxioImager and AxioCam RMC (Zeiss, Germany), using the Dye Alexa fluor 488 filters (Fluorescein and alexa 488 (450/490–515 nm), Filter set 09 (488009-9901-000) and the Dye DAPI filter (DAPI (365 nm–420 nm), Filter set 02 (488002-9901-000).

### 3D modeling of adult worm *Trichuris muris* attachment to the intestinal mucosa and bacterial translocation

Two chemically fixed intestines were washed in PBS, stained with 500 μM calcein for 15 minutes, and then washed in pure water before being mounted in capillary glass with 1.0% agarose. The samples were imaged via a Zeiss Lightsheet Z.1 microscope (Zeiss, Germany) equipped with a 498 nm laser and a Plan-Neofluar 5x/0.16 objective at CENABIO-UFRJ. Five chemically fixed intestines were stained with a 2% aqueous solution of osmium for 12hrs followed by incubation for 6 days in PMA or PTA, placed in a plastic tube containing water, and scanned via the nato/micro-CT GE Phoenix v|tome|x 240 (Baker Hughes, USA) at the CDB at USP-Ribeirão Preto. The acquisition settings were as follows: accelerating voltage of 50 kV at 200 µA, average of 4 images/projection with 333 ms/image, 1,500 projections over 360°, binning of 1, and voxel size ranging from 6.7 to 10.3 µm depending on magnification. Stacked images were aligned via IMOD [27], and a preliminary 3D model was generated via AMIRA software (Thermo-Fisher). Blender, a free and open-source 3D modeling software, was used to refine the 3D model and render an animation of the parasite‒host interaction. Three-dimensional printed models of the parasite and intestine scaled at 20 times the real size were produced using two types of 3D printers. For the intestinal tissue, a fused deposition modeling (FDM) printer, model Ender 3 Pro from Creality, and a pink-colored PLA filament were used. For the nematodes, a resin 3D printer model Anycubic Mono X 4K with clear resin was used, both of which were installed at SAUDE3D-UERJ. The models were then glued together to represent the interaction between the parasite and the host.

### Statistical analysis

Differences among experimental groups in all the experiments were assessed using Student’s t test, with a significance of P ≤ 0.05. All the data were analyzed via GraphPad Prism (version 5) software and GraphPad InStat software (GraphPad, USA).

## Results

### Investigating the ultrastructure of intestinal damage caused by *Trichuris muris* larvae during the acute phase of infection

**Fig 1:**
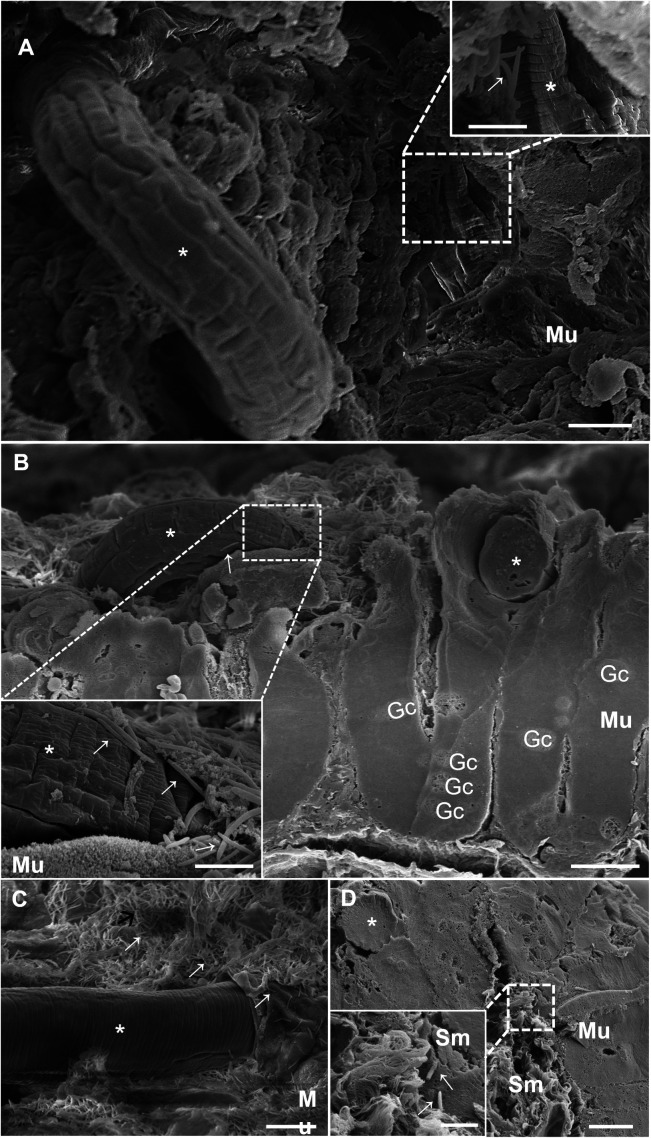
SEM of the cecum with *Trichuris muris* disrupting the intestinal mucosa after 10 and 17 days of infection and bacterial adhesion. (A) SEM image of the cecum of mice infected with *Trichuris muris* 10 days after infection. (B, C and D) SEM image of the cecum of a mouse infected with *Trichuris muris* for 17 days. (A) SEM image of the intestinal mucosa and the route taken by the parasite (asterisk) that subsequently penetrates into the mucosa and breaks parts of the intestinal mucosa, with bacteria close to the rupture site (dotted square). The inset shows the parasite penetrating the tissue at higher magnification and carrying bacteria (arrow) present on the mucosal surface (Mu), revealing the path taken by the parasite in the intestinal mucosa. The scale bars represent 10 µm; (inset) 5 µm. (B) Cecum fracture showing the intestinal mucosa and the parasite (asterisk) inserted, forming the syncytial tunnel, goblet cells (Gc), rupturing the intestinal mucosa, and carrying bacteria (arrows) in the mucosa. The scale bar represents 20 µm. The inset shows the parasite (asterisk) penetrating the mucosa and carrying bacteria (arrows) into the mucosa at higher magnification. Scale bar represents 5 µm. (C) Parasite (asterisk) inserted in the intestinal mucosa with bacteria adhered (arrow) to its body and to the surface of the intestinal mucosa. The scale bar represents 20 µm. (D) Cecum fracture showing the nematode (asterisk) and mucosa (Mu) and submucosa (Sm) layers with bacteria in the submucosa (arrows). The scale bar represents 20 µm. Insets of the invasive bacteria (arrows) inside the submucosa (Sm). The scale bar represents 5 µm.

**Fig 2:**
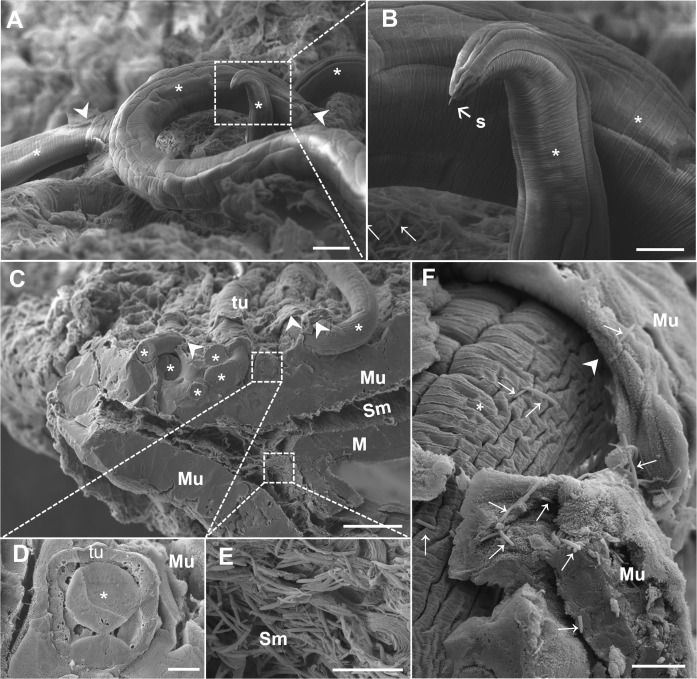
SEM of the cecum with *Trichuris muris* disrupting the intestinal mucosa after 17 and 22 days of infection and bacteria invading the submucosa. (A and B) SEM image of the cecum of a mice infected with *Trichuris muris* for 17 days. (C, D, E and F) SEM image of the cecum of a mouse infected with *Trichuris muris* for 22 days. (A) Parasite (asterisk) penetrating into the intestinal mucosa, causing ruptures (arrowhead), breaking several regions of tissue during migration throughout the syncytial tunnel. Scale bar represents 30 µm. (B) Anterior end of the nematode showing the stylet (s) and bacteria on the mucosa surface (arrows). Scale bar represents 10 µm. (C) Fractured cecum showing the three layers of the mucosa (Mu), submucosa (Sm) and muscular layer (M), and the parasite (asterisk) penetrating into the mucosa, forming the syncytial tunnel (tu) and causing ruptures (arrowhead) on the tissue surface and high bacterial levels in the submucosa. The scale bar represents 100 µm. (D) Details of the parasite (asterisk) inside the tunnel (tu) in a cross-section of mucosa (Mu). Scale bar represents 10 µm. (E) Details of the bacteria inside the intestinal submucosa (Sm). Scale bar represents 10 µm. (F) Detail of the parasite (asterisk) breaking the intestinal mucosa (Mu); the arrowheads indicate bacteria adhering to the nematode cuticle and on the microvilli of the intestinal mucosa (arrows). Scale bar represents 10 µm.

After 10 days of infection, L2 larvae were observed breaking the epithelial surface. The presence of bacteria close to the nematode cuticle and rupturing were commonly observed (Fig 1A; Inset). In a transverse fracture of the cecum, at 17 d.p.i., larvae from the L3 larval stage were penetrating into the intestinal mucosa, and a large number of bacilliform bacteria were present on the mucosa surface and adhered to the nematode cuticle (Fig 1B and 1C). In Fig 1C, the larva is located in the mucosa, inserted, close to a region highly populated with bacteria. At this stage, we observed the nematode larvae inside the syncytial tunnel within the epithelial cells at the surface of the mucosa adjacent to the lumen (Fig 1B) and the presence of bacterial structures inside the tissue in the submucosal layer (Fig 1D; Inset). Additionally, we observed the specific moment at which the anterior region pointed toward the intestinal surface, which was evidence of the initial movement of the head-down behavior before new penetration of the tissue (Fig 2A and 2B). At the anterior end of the nematode, it was possible to identify the extroverted stylet that can be used by the nematode to emerge from the tunnel and generate a new perforation into the tissue. Together, these results strongly suggest a dynamic association between the attachment and movement of the parasite and bacterial translocation.

At 22 d.p.i., we observed fractures in the cecum, revealing the mucosa, submucosa and muscle layers, with L4 larvae causing several ruptures in the mucosa (Fig 2C) and penetrating into the syncytial tunnels (Fig 2D). Furthermore, many bacilliform bacteria were present in the mucosa, forming an agglomerate structure in the submucosa (Fig 2E). In addition, spaces between the nematode and the tunnel borders can be observed, revealing the presence of bacteria adhering to the larval cuticle (Fig 2F). The identification of a large number of bacteria in the submucosa at 22 d.p.i. during the acute phase of infection coincided with the high temperatures observed in the infected mice compared with those in the noninfected group (S1 Fig). The damage caused by the parasite creates spaces and chambers that may represent areas with low or no oxygen, promoting the development of anaerobic bacteria.

### Alterations in the peripheral blood, peritoneal cells and immune system associated with intestinal damage

**Fig 3:**
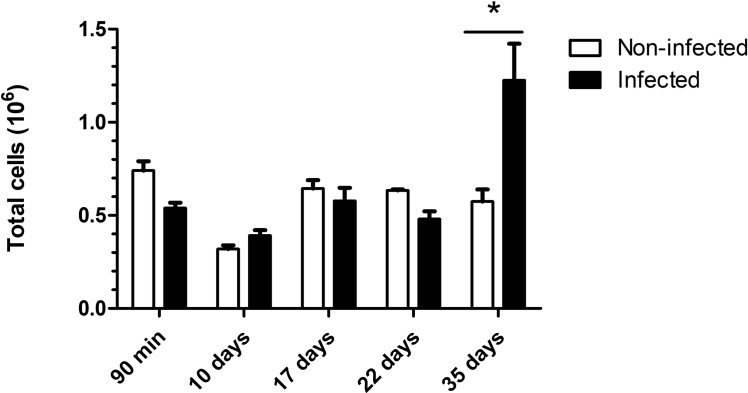
Total number of peritoneal cells. Variation in the total number of peritoneal cells observed in the *Trichuris muris* infection group. The significance of differences between the noninfected and infected groups (n = 5) was determined via Student’s t test, and asterisks indicate statistically significant differences. * p value ≤ 0.05.

**Fig 4:**
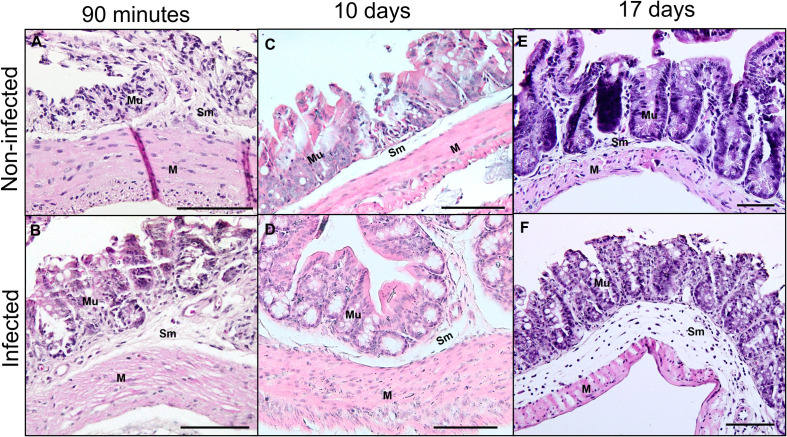
Histology of the cecum after 90 minutes and 10 and 17 days of infection by *Trichuris muris.* Histological section of the large intestine (cecum) stained with hematoxylin and eosin showing the morphology and morphometry of the large intestine layers. (A, C, E) Group not infected and (B, D, F) infected with *Trichuris muris*. (A, B) 90 minutes after infection. (C, D) 10 days after infection. (E, F) 17 days after infection. An increase in the thickness of the mucosa (Mu), submucosa (Sm) and muscle layers (M) and increased infiltration of inflammation in the submucosa were observed beginning on the 10th day of infection. Scale bar represents 100 µm.

**Fig 5:**
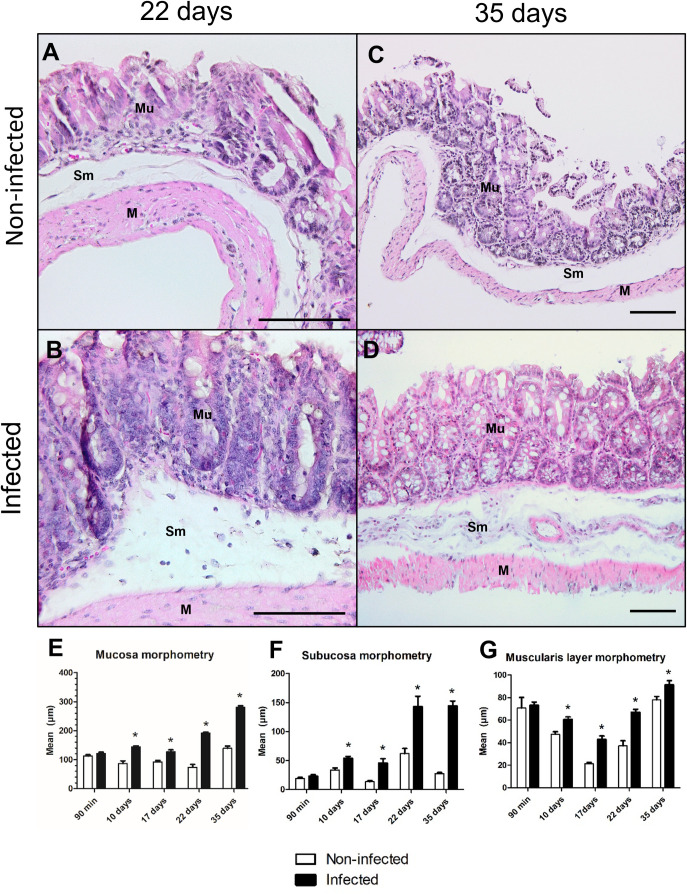
Histology of the cecum after 22 and 35 days of infection by *T. muris.* Histological section of the large intestine (cecum) stained with hematoxylin and eosin showing the morphology and morphometry of the large intestine layers. (A, C) Noninfected group; (B, D) infected with *Trichuris muris*. (A, B) After 22 days of infection. (C, D) After 35 days of infection. (E) Morphometric analyses of the mucosa (Mu); (F) morphometric analyses of the submucosa (Sm); (G) morphometric analyses of the muscularis layers (M), showing an increase from the 10th day of infection to the 35th day. The significance of differences between the noninfected and infected groups (n = 10) was determined via Student’s t test, and asterisks indicate statistically significant differences. * p value ≤ 0.05. Scale bar represents 100 µm.

Chronic infection with *T. muris* causes rigidity in the cecum and significant thickening of the mucosa, submucosa, and muscularis layers, mainly due to inflammatory infiltration [3]. In our study, we observed alterations in blood cells during the acute phase of infection, with a decrease in the number of circulating monocytes and an increase in the number of eosinophils at 10 d.p.i.. This increase in eosinophils was maintained at other time points measured during infection evolution (17^th^, 22^nd^, and 35^th^ days) (S1 Table). Additionally, a significant increase in basophils was noted at 22 d.p.i.. The total number of peritoneal cells was greater in the group infected only at 35 d.p.i. (Fig 3). This shows a relative systemic balance of the infection based on observations of the gradual growth of inflammatory infiltrate cells in the cecum, which begins in the acute phase (10 d.p.i.) and extends until the chronic phase (35 d.p.i.), causing a thickening of the mucosa, submucosa and muscular layer (Figs 4 and 5). This finding suggests that the presence of the nematode and bacterial translocation initially cause an imbalance in the intestine, leading to the release of soluble antigens, which subsequently impact other tissues and organs.

**Fig 6:**
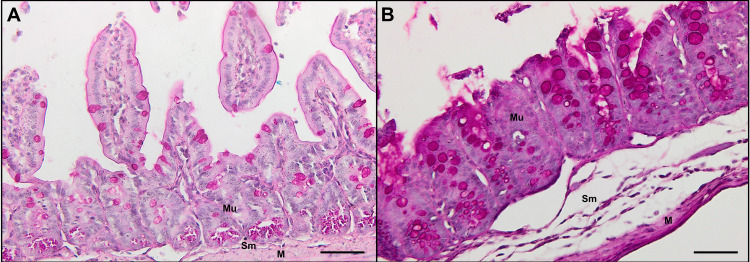
Increased number of goblet cells and enterocytes from the 17th day of infection. Histological sections of the large intestine (cecum) of 17-day-infected mice stained with PAS. (A) Noninfected mice showing mucosa (Mu) with few goblet cells and enterocytes (cells color pink), thin submucosa (Sm) and a muscle layer (M). (B) Mice infected with *Trichuris muris* presented an increase in the number of goblet cells and enterocytes (pink) in the mucosa (Mu), submucosa (Sm) and muscular layer (M). Scale bar represents 50 µm.

The increase in the thickness of the intestinal layers observed in the infected group compared with the respective uninfected group is attributed to the increase in different intestinal and immune cells. Morphological analysis of the intestinal mucosa revealed an increase in the number of eosinophils and neutrophils starting at 10 days and persisting throughout the acute phase, up to the 22^nd^ day after infection. An increase in the number of lymphocytes was observed at 17 days, and both lymphocytes and macrophages increased at 22 days in the acute phase. In the chronic phase, at 35 days, an increase in the number of eosinophils and macrophages was observed (Table 1). In the submucosal analyses, all the cell counts increased starting at 10 days after infection, persisting throughout the acute and chronic phases (Table 2). With respect to the quantification of enterocytes and goblet cells, we observed a significant increase in the number of these cells starting at the 10^th^ and 17^th^ days after infection, respectively, and remaining throughout the acute and chronic phases (Fig 6 and Table 3).

**Table 1:** Intestinal mucosa morphological analysis.

		Eosinophil (Mean ± ErroPad)	Macrophages (Mean ± ErroPad)	Neutrophil (Mean ± ErroPad)	Lymphocytes (Mean ± ErroPad)
**90 minutes**	Noninfected	0.17 ± 0.17	5.17 ± 1.08	2.67 ± 0.42	1.33 ± 0.33
	Infected	0.17 ± 0.17	7.83 ± 1.25	2.83 ± 0.79	1.00 ± 0.26
	p value	p > 0.999	p = 0.1371	p = 0.8564	p = 0.4475
**10 days**	Noninfected	0.17 ± 0.17	9.00 ± 1.32	3.17 ± 0.54	1.17 ± 0.31
	Infected	3.17 ± 0.70	12.5 ± 1.20	5.83 ± 0.91	2.83 ± 0.70
	p value	p = 0.0020*	p = 0.0782	p = 0.0305*	p = 0.0550
**17 days**	Noninfected	0.17 ± 0.17	7.33 ± 1.38	2.28 ± 0.41	1.94 ± 0.36
	Infected	6.50 ± 1.12	15.67 ± 0.80	4.67 ± 0.63	3.83 ± 0.37
	p value	p = 0.0003*	p = 0.0004*	p = 0.0032*	p = 0.0010*
**22 days**	Noninfected	1.39 ± 0.39	4.06 ± 0.88	2.39 ± 0.37	1.83 ± 0.35
	Infected	3.00 ± 0.43	6.83 ± 1.21	3.94 ± 0.54	3.50 ± 0.42
	p value	p = 0.0086*	p = 0.0717	p = 0.0233*	p = 0.0047*
**35 days**	Noninfected	1.39 ± 0.39	5.67 ± 1.43	4.67 ± 1.05	3.50 ± 0.89
	Infected	3.06 ± 0.43	12.83 ± 1.74	6.33 ± 1.20	4.50 ± 0.50
	p value	p = 0.0070*	p = 0.0098*	p = 0.3217	p = 0.3484

The significance of differences between the noninfected and infected groups (n = 6) was determined via Student’s t test, and asterisks indicate statistically significant differences.

* p value ≤ 0.05.

**Table 2:** Intestinal submucosa morphological analysis.

		Eosinophil (Mean ± ErroPad)	Macrophages (Mean ± ErroPad)	Neutrophil (Mean ± ErroPad)	Lymphocytes (Mean ± ErroPad)
**90 minutes**	Noninfected	0.17 ± 0.17	6.67 ± 1.17	2.17 ± 0.40	2.67 ± 0.33
	Infected	0.17 ± 0.17	6.67 ± 0.72	2.50 ± 0.56	2.33 ± 0.92
	p value	p > 0.9999	p > 0.9999	p = 0.6400	p = 0.7402
**10 days**	Noninfected	0.17 ± 0.17	4.00 ± 1.03	1.33 ± 0.42	0.83 ± 0.17
	Infected	1.33 ± 0.42	10.67 ± 1.76	3.00 ± 0.37	2.50 ± 0.34
	p value	p = 0.0277*	p = 0.0086*	p = 0.0136*	p = 0.0014*
**17 days**	Noninfected	0.50 ± 0.22	5.17 ± 0.91	1.50 ± 0.43	0.83 ± 0.31
	Infected	4.33 ± 1.56	9.17 ± 1.52	6.17 ± 1.14	3.50 ± 0.81
	p value	p = 0.0356*	p = 0.0471*	p = 0.0033*	p = 0.0114*
**22 days**	Noninfected	0.17 ± 0.17	3.17 ± 0.31	1.50 ± 0.43	0.67 ± 0.21
	Infected	2.67 ± 0.72	9.50 ± 1.09	5.00 ± 0.86	3.50 ± 0.34
	p value	p = 0.0067*	p = 0.0002*	p = 0.0044*	p < 0.0001*
**35 days**	Noninfected	0.17 ± 0.17	2.50 ± 0.43	1.17 ± 0.48	0.33 ± 0.21
	Infected	4.50 ± 0.67	13.67 ± 1.28	9.67 ± 1.15	8.33 ± 1.75
	p value	p < 0.0001*	p < 0.0001*	p < 0.0001*	p = 0.0011*

The significance of differences between the noninfected and infected groups (n = 6) was determined via Student’s t test, and asterisks indicate statistically significant differences.

* p value ≤ 0.05.

**Table 3:** Quantification of goblet cells and enterocytes.

		90 min (Mean ± ErroPad)	10 days (Mean ± ErroPad)	17 days (Mean ± ErroPad)	22 days (Mean ± ErroPad)	35 days (Mean ± ErroPad)
Goblet cells	Noninfected	74.75 ± 2.81	64.50 ± 4.87	48.50 ± 6.70	47.0 ± 7.65	35.50 ± 5.50
	Infected	54.50 ± 1.71	38.67 ± 1.22	114.0 ± 25.03*	111.67 ± 19.79*	109.50 ± 24.29*
	p value	p = 0.4100	p = 0.0659	p = 0.0448	p = 0.0187	p = 0.0249
Enterocytes	Noninfected	51.69 ± 4.79	62.56 ± 2.02	59.38 ± 2.36	51.06 ± 4.64	50.63 ± 2.84
	Infected	52.81 ± 4.34	83.94 ± 4.10*	79.88 ± 2.18*	86.00 ± 2.82*	83.88 ± 3.87*
	p value	p = 0.8631	p < 0.0001	p < 0.0001	p < 0.0001	p < 0.0001

The significance of differences between the noninfected and infected groups (n = 4) was determined via Student’s t test, and asterisks indicate statistically significant differences.

* p value ≤ 0.05.

**Fig 7:**
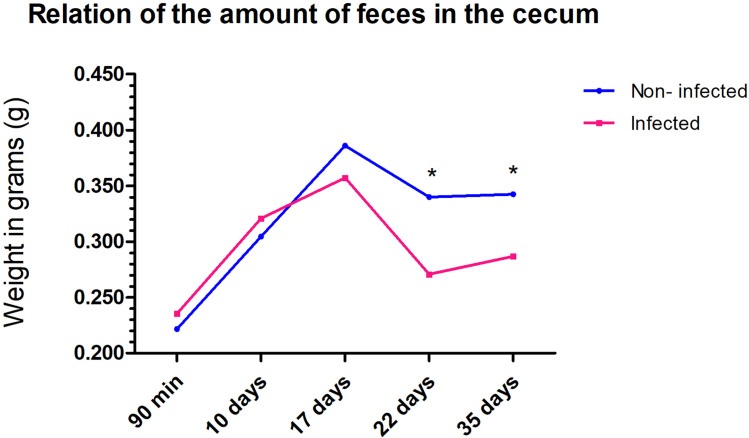
Quantification of the amount of feces. Graph illustrating the percentage of feces retained in the cecum of the mice. Between the 22^nd^ and 35^th^ days of infection, fewer feces were retained in the cecum of the mice infected with *Trichuris muris*. The significance of differences between the noninfected and infected groups (n = 5) was determined via Student’s t test, and asterisks indicate statistically significant differences. * p value ≤ 0.05.

On the basis of our results, the rigidity of the cecum observed in the chronic phase begins in the acute phase of infection, with swelling of the organ resulting from cellular infiltration, hyperplasia, and hypertrophy of the tissues. Furthermore, the inflammatory process can impact the peristaltic function of the organ. This disturbance was evident when we measured and compared the quantity of feces present in the cecum of infected and noninfected mice. Although it did not impact the animals’ body weight (S2 Table), this intestinal infection impacted the storage capacity of the fecal contents, which could affect the digestive process and the nutritional profile of the infected hosts (Fig 7).

### Acute infection impacts the immune response in the cecum, lymph nodes, peritoneal macrophages, and spleen

**Fig 8:**
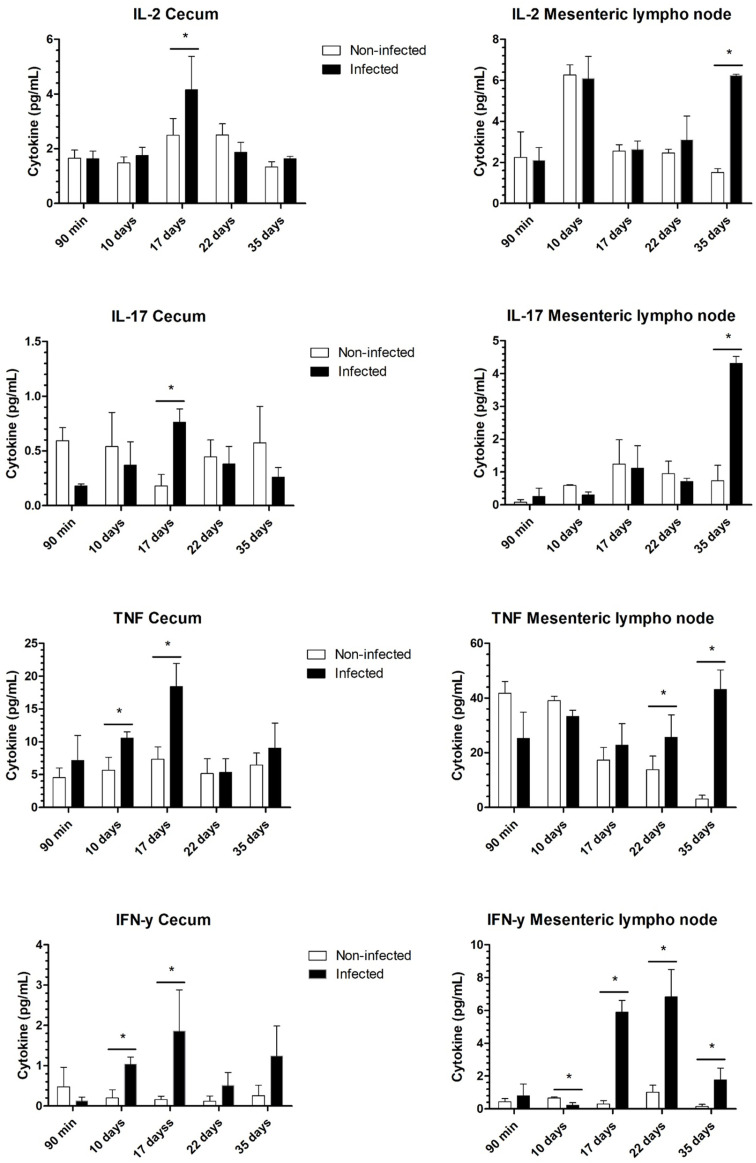
Th1 profile of cytokines present in the cecum and mesenteric lymph nodes throughout infection. Cytokine Th1 secretion in the cecum and mesenteric lymph nodes of noninfected and infected mice by *Trichuris muris*. The results are expressed in picograms per milliliter. After isolation and homogenization, the proteins were measured via an ELISA kit. The significance of differences between the noninfected and infected groups (n = 4) was determined via Student’s t test, and asterisks indicate statistically significant differences. * p value ≤ 0.05.

**Fig 9:**
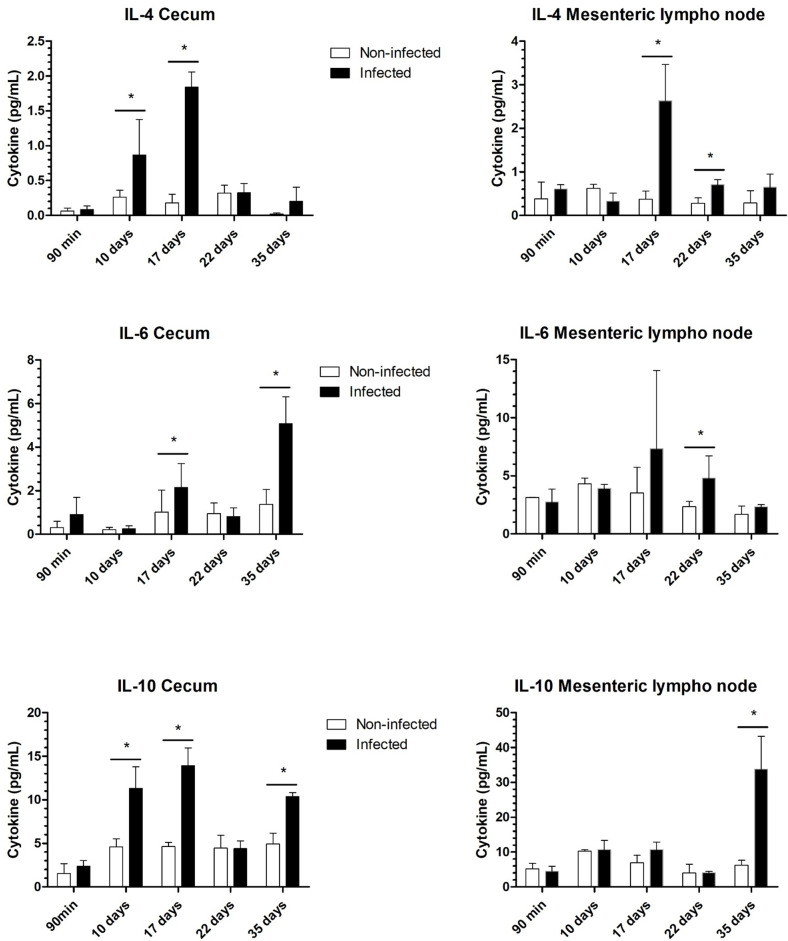
Th2 profile of cytokines present in the cecum and mesenteric lymph nodes throughout infection. Cytokine Th2 secretion in the cecum and mesenteric lymph nodes of noninfected and infected mice by *Trichuris muris*. The results are expressed in picograms per milliliter. After isolation and homogenization, the cytokines were measured via an ELISA kit. The significance of differences between the noninfected and infected groups (n = 4) was determined via Student’s t test, and asterisks indicate statistically significant differences. * p value ≤ 0.05.

In the analysis of cytokines in the infected cecum, site of worm insertion, we observed a significant increase in IL-4, IL-10, TNF, and IFN-γ levels 10 days after infection. By 17 days, increases in the levels of IL-2, IL-4, IL-6, IL-10, IL-17, TNF, and IFN-γ were noted in the infected groups compared with those in the noninfected groups. However, at 22 days after infection, we did not observe differences in the cytokines secreted by cecum cells between the infected and noninfected groups. At 35 days after infection, during the chronic phase, we observed an increase in the Th2 cytokines IL-6 and IL-10 in infected mice (Figs 8 and 9). Importantly, the molting of L3 larvae to L4 larvae occurs at 22 days, and parasite development is associated with the maturation of the bacillary glands, which are fully developed in adult worms and associated with immunomodulatory products.

**Fig 10:**
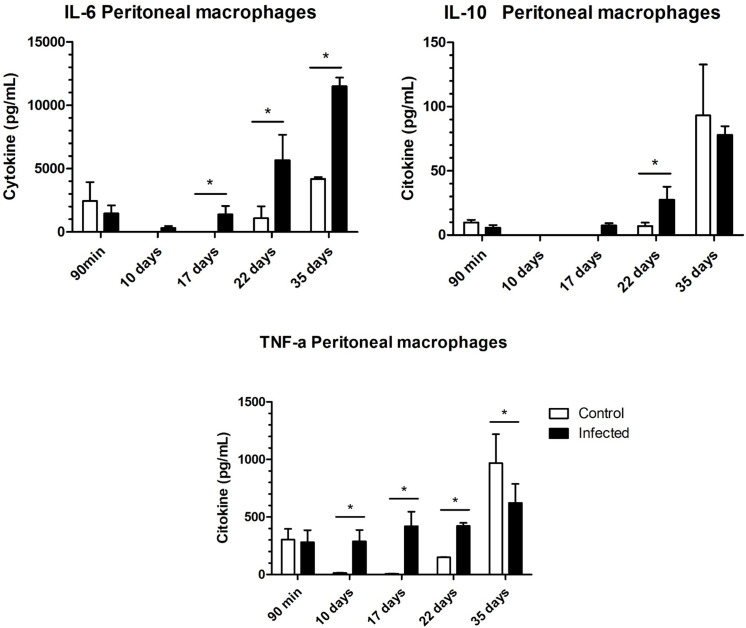
Cytokine secretion in peritoneal macrophages. Cytokine secretion in peritoneal macrophages of noninfected and infected mice by *Trichuris muris*. After isolation and homogenization, the cytokines were measured via an ELISA kit. The significance of differences between the noninfected and infected groups (n = 4) was determined via Student’s t test, and asterisks indicate statistically significant differences. * p value ≤ 0.05.

**Fig 11:**
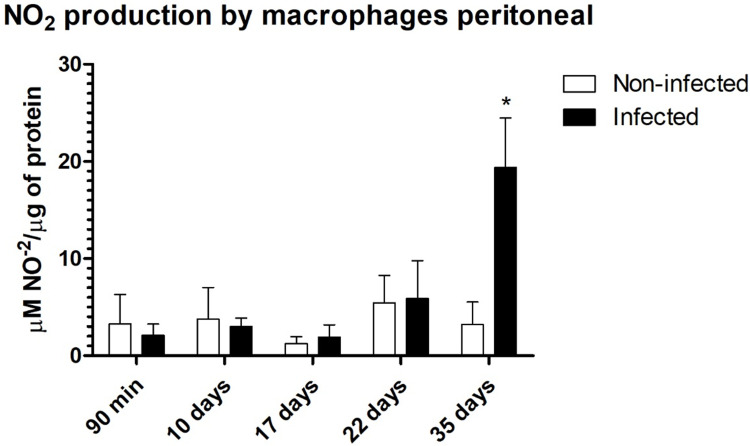
Nitric oxide dosage of peritoneal macrophages. Cultures of peritoneal macrophages from noninfected and infected *Trichuris muris*-infected mice were maintained under culture conditions for 24h and stimulated with LPS (3 g/ml), and NO-2 was measured. The significance of differences between the noninfected and infected groups (n = 4) was determined via Student’s t test, and asterisks indicate statistically significant differences. * p value ≤ 0.05.

On the other hand, in the lymph node and macrophage analyses, a mixed immune response was observed only in the later stage of the acute phase (22 days after infection) and in the chronic phase. These findings indicate that fourth-stage larvae and adult worms may modulate the immune response at the site of infection, as previously mentioned. However, a mixed response is found in other anatomical locations. In the lymph nodes, we observed significant increases in the levels of IL-4, IL-6, TNF, and IFN-γ at 22 days after infection and increases in the levels of IL-2, IL-1, and IL-10 in infected mice at 35 days (Figs 10 and 11). In the analysis of cytokines produced by peritoneal macrophages, we observed increases in TNF-α, IL-6, and IL-10 after 22 days (Fig 10). Additionally, there was an increase in nitric oxide produced by these macrophages on the 35th day of infection when stimulated by LPS, indicating their activation (Fig 11). We did not observe differences in MCP-1 levels between the groups (S2 Fig), and IL-12p70 levels were undetectable in all analyses.

**Fig 12:**
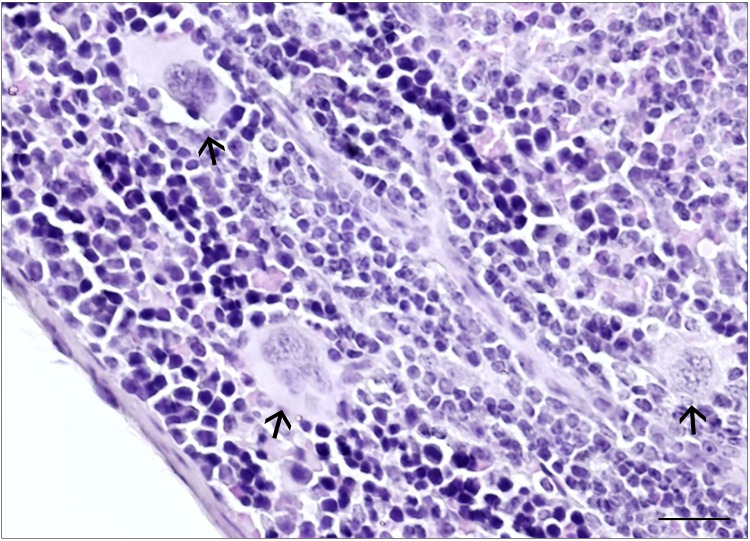
Spleen histology showing megakaryocytes. Histological section of the spleen, which was stained with hematoxylin and eosin from a 35-day-old mouse infected with *Trichuris muris*, showing megakaryocytes (arrow). Scale bar represents 20 μm.

At the end of the acute phase and during the chronic phase of infection, on the 22nd day, we observed a greater spleen weight in the infected group than in the noninfected group. Additionally, at 22 and 35 days after infection, the spleens of infected mice were longer than those of noninfected mice. Morphometric analysis of the white pulp revealed a larger area in infected mice at 17, 22, and 35 days of infection. Moreover, at 35 days, the red pulp contained a greater number of megakaryocytes (Fig 12 and Table 4).

**Table 4:** Spleen data obtained throughout the infection.

		90 min	10 days	17 days	22 days	35 days
Spleen weight (g)	Noninfected	0.05 ± 0.01	0.07 ± 0.01	0.07 ± 0.01	0.07 ± 0.01	0.11 ± 0.02
	Infected	0.07 ± 0.01	0.06 ± 0.01	0.07 ± 0.01	0.10 ± 0.01	0.11 ± 0.01
	p value	p = 0.2480	p = 0.2181	p = 0.9363	p = 0.0888	p = 0.8911
Spleen weight in relation to body weight (%)	Noninfected	0.26 ± 0.05	0.36 ± 0.02	0.31 ± 0.01	0.28 ± 0.02	0.44 ± 0.08
	Infected	0.35 ± 0.05	0.34 ± 0.01	0.32 ± 0.02	0.44 ± 0.04*	0.44 ± 0.02
	p value	p = 0.0645	p = 0.3450	p = 0.4742	p = 0.0197	p = 0.9060
Spleen size (mm)	Noninfected	12.10 ± 0.90	12.89 ± 0.24	13.59 ± 0.26	13.55 ± 0.11	13.60 ± 0.20
	Infected	12.50 ± 0.47	13.03 ± 0.25	13.10 ± 0.24	15.17 ± 0.42*	15.13 ± 0.22*
	p value	p = 0.6889	p = 0.7046	p = 0.2035	p = 0.0043	p = 0.0054
Size of the white pulp (µm)	Noninfected	97,799 ± 12,874	140,430 ± 38,372	48,456 ± 4,157.3	52,010 ± 7,948.5	125,659 ± 36,966
	Infected	124,672 ± 14,100	108,751 ± 75,457	97,359 ± 18,497*	110,657 ± 24,721*	102,392 ± 6,021.5*
	p value	p = 0.1651	p = 0.3629	p = 0.0004	p = 0.0162	p = 0.5272
Number of megakaryocytes	Noninfected	3.25 ± 0.95	1.67 ± 0.67	1.75 ± 0.48	2.25 ± 0.48	1.50 ± 0.29
	Infected	5.00 ± 1.23	3.00 ± 0.71	3.25 ± 1.32	2.00 ± 0.41	3.25 ± 0.63
	p value	p = 0.3014	p = 0.2422	p = 0.3250	p = 0.7049	p = 0.0448*

The significance of differences between the noninfected and infected groups (n = 5) was determined via Student’s t test, and asterisks indicate statistically significant differences.

* p value ≤ 0.05.

### Mapping of the translocation process via *in situ* hybridization

**Fig 13:**
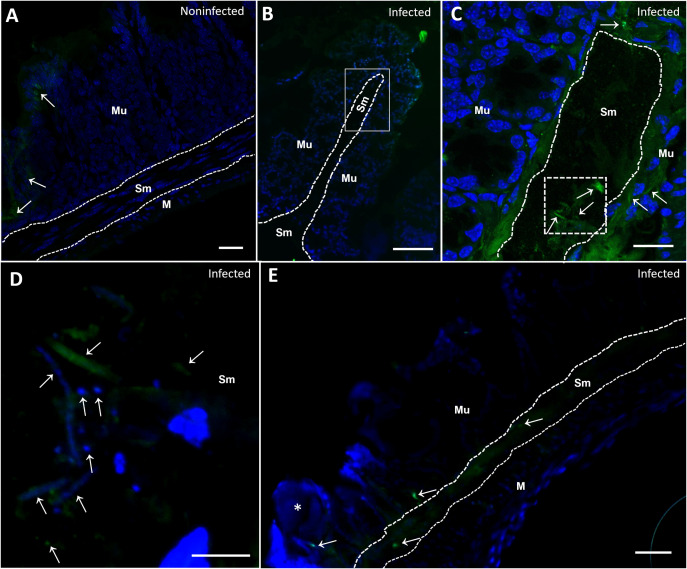
Fluorescence *in situ* hybridization (FISH) of sections from noninfected and *Trichuris muris*-infected mice revealed bacterial invasion beginning on the 17th day of infection. Merged images showing bacteria (green) and host tissue cells (DAPI). (A) Noninfected mouse cecum after 35 days, showing three tissue layers, the mucosa (Mu), submucosa (Sm) and muscle layers (M), with bacteria found only in the intestinal mucosa layer. Scale bar represents 20 µm. (B) Mouse cecum after 17 days of infection, showing the intestinal mucosa (Mu) and submucosa (Sm) layers and bacterial invasion in the submucosa. Scale bar represents 20 µm. (C) The inset of (B) shows the mucosa (Mu) and submucosa (Sm), with bacteria (arrow) in the submucosa. Scale bar represents 10 µm. (D) The inset of (C) is shown at higher magnification, with the submucosa with coccus and bacillus bacteria (arrow) invading the tissue, as indicated by the arrow. Scale bar represents 5 µm. (E) Mouse cecum after 22 days of infection showing the parasite (asterisk) inserted into the intestinal mucosa (Mu), bacteria (arrow) close to the parasite and invading the intestinal submucosa (Sm), and the muscle layer is large. Scale bar represents 10 µm.

After performing scanning electron microscopy and assessing cytokine dynamics, we conducted fluorescence *in situ* hybridization (FISH) experiments to determine the timing of bacterial translocation during the acute phase of infection. The invasive process was confirmed to start at 17 days after infection. In the cecum of the mice in the noninfected group, bacteria (green) were present only in the mucosa layer and in the crypts of Lieberkühn (Fig 13A). However, in samples from infected mice, bacteria were identified inside the submucosa (Fig 13B, 13C and 13D) at the 17^th^ and 22^nd^ (Fig 13E) days after infection (acute phase) and, as expected, after 35 days of infection, in the chronic phase.

### Visualization of adult worm attachment mechanisms and implications for bacterial translocation: Insights from 3D modeling and animation

**Fig 14:**
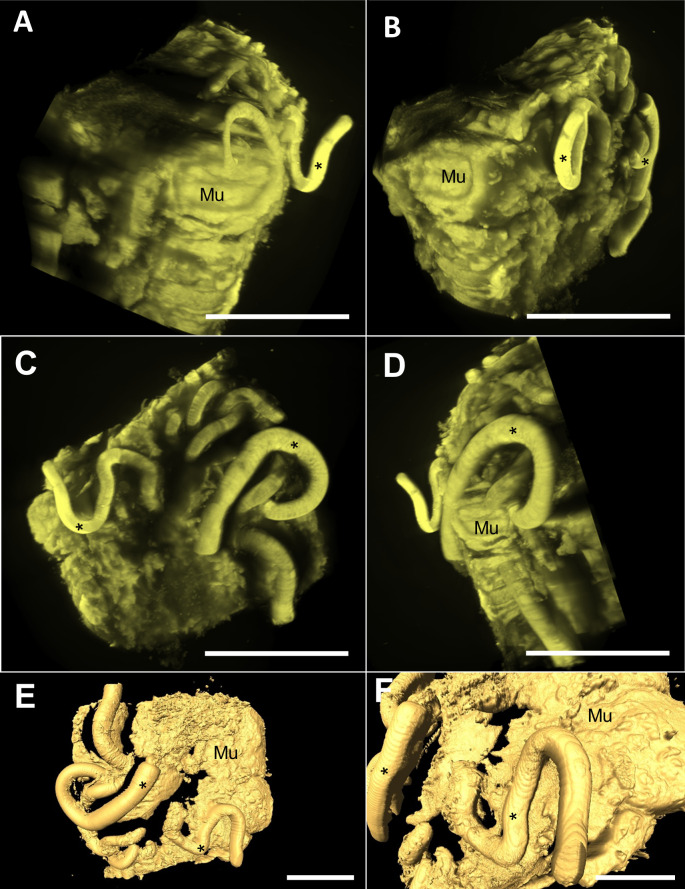
Images of the 3D model obtained via light sheet microscopy showing the infected intestine in the chronic phase of infection. (A–D) Series of the infected intestine model with transparency performed via Zen software, showing different angles of the mucosa (Mu) and nematode (asterisk). Scale bar represents 10 µm. (E, F) Three-dimensional model performed via Amira’s automatic segmentation isosurface showing the nematode (asterisk) and mucosa surface (Mu). Scale bar represents 300 µm.

To investigate the attachment process of the adult worm to the host intestine and assess its influence on bacterial translocation, we used lightsheet microscopy and X-ray microtomography (micro-CT) integrated with scanning electron microscopy (SEM). Light sheet microscopy provides simultaneous surface and volume information of both the parasite and the intestinal tissue, raising new questions about the complexity of this attachment process. This includes the mechanical action of the nematode, which creates new spaces in the intestinal epithelium that may favor the development of anaerobic bacteria. The 3D models highlighted the different points of insertion and attachment of the nematode on the mucosal tissue surface (Fig 14).

**Fig 15:**
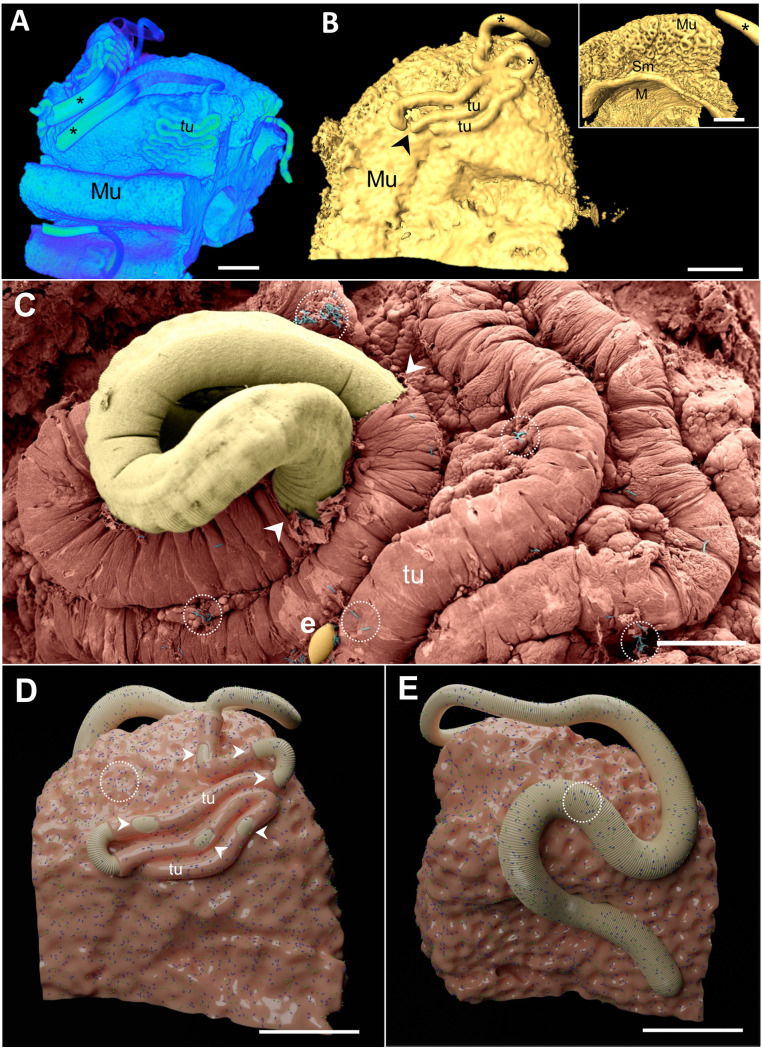
Three-dimensional modeling and color scanning electron microscopy (SEM) results were integrated to explain the bacterial translocation process. (A) Micro-CT image of the cecum ofa 35-day-old mouse showing epithelial tunnels (tu) in different parts of the surface mucosa (Mu) and the posterior region of the parasite in the lumen (asterisk). Scale bar represents 500 µm. (B) Three-dimensional model based on Amira’s automatic segmentation isosurface showing the penetrating nematode (tu), tissue breaks (arrowhead), and posterior region of the parasite (asterisk). Inset lateral view of the model showing the three intestinal layers, including the mucosa (Mu), submucosa (Sm), muscular layer (M), and the nematode (asterisk). The scale bar represents 1 mm. Inserted (B) images showing the mucosa (Mu), submucosa (Sm) and muscle layer (M) at lower magnification and parasites in the lumen. The scale bar represents 1 mm. (C) SEM image of the mucosa surface (pink color) showing the anterior region of the nematode (yellow color), tissue breaks on the mucosa (arrowhead) exposing the nematode cuticle, nematode egg on the surface (e), and bacteria (blue color – dotted circles). The scale bar represents 100 µm. (D, E) Rendered three-dimensional models integrating results and showing the nematode (yellow color), tissue breaks (arrowheads), epithelial tunnels (tu), and bacteria (green and purple - dotted circles) on the mucosal surface (pink) and on the nematode body. Scale bars represent 500 µm.

Our micro-CT results of the infection site revealed the organization of the adult worm attached to the host intestine, including the formation of epithelial tunnels covering the anterior region of the nematode and tissue breaks that exposed the nematode body (Fig 15A and 15B). The integrated SEM images (Fig 15C) revealed that bacteria on the mucosal surface adhered to the nematode cuticle (S3 Fig). These findings, along with the rendered models, were used to create an animation illustrating the dynamic relationship of the parasite with the host tissue and intestinal bacteria (Fig 15D and 15E). The loose geometry was deleted, and the main part of the model was chosen for further refinement. The surface of the intestine was smoothed with Blender’s subdivision surface and smooth modifiers, which enabled the model to be carved and enhanced via the software’s sculpting mode. Blender digital modeling tools were also used to reconstruct helminths and determine their characteristic surface textures. Using the rendering features inside Blender, it was possible to apply color and visual texture to both models and later sprinkle bacteria shaped objects along the intestine and helminth, simulating bacterial invasion when animated. The parasite was also animated via paths to simulate the movement and tears made during this process, revealing how nematode movement promotes bacterial translocation from the intestinal lumen to underlying tissues, such as the lamina propria and submucosa (S1 Movie). Three-dimensional printed models of the parasite and intestine were scaled to 20 times the actual size via two types of 3D printers. The model details the mucosa surface, the crypts of Lieberkuhn depressions, epithelial breaks, and the nematode surface with a texture such as transverse cuticular striation. The model provides topographic details, tactile texture and similar colors observed in *in vivo* samples for in-class and outreach showcasing (S4 Fig).

## Discussion

Chronic infection leads to mucosal lesions, facilitating bacterial translocation from the intestinal lumen to the submucosa, potentially involving pathogenic *Escherichia coli* strains associated with inflammatory bowel diseases [28]. This bacterial invasion triggers an inflammatory response, leading to tissue thickening, goblet cell proliferation, and blood parameter alterations [3]. Trichuriasis disrupts the intestinal microbiota, particularly affecting *Bacteroides* and *Lactobacillus* populations, regardless of the parasite infection load, whether at low-doses without immunomodulation or at high doses [3,29,30]. Even after parasite elimination, bacterial translocation may contribute to chronic inflammatory conditions. While whipworms depend on the host’s microbiota for efficient hatching of eggs, their presence in the intestine alters the local microbiome, inhibiting the hatching of new parasite eggs and thus protecting the host against overpopulation [31].

L1 stage larvae remain entirely submerged in the tissue, creating “multiple intracellular tunnels” that cause minimal damage to the host cells [13]. These findings explain why, after 90 minutes of infection, we did not observe local or systemic alterations in the infected group of mice. On the other hand, our scanning electron microscopy (SEM) results revealed that in the acute phase, at 10 days of infection, the parasite, which is already in the L2 larval stage, is capable of breaking the intestinal tissue covering, creating the first channels for the intestinal microbiota to reach subjacent tissues. This impacts the host’s homeostasis due to the presence of parasite and bacterial antigens and tissue rupture, releasing alarmins (PAMPs: Pathogen-associated molecular patterns. DAMPs: Molecules present in damaged cells) and chemokines, which trigger the recruitment of inflammatory cells to the cecum, the specific infection site of this nematode [32]. Moreover, [33] and [13] described the displacement and anchoring mechanism of *T*. *muris* adult worms, suggesting, on the basis of different images obtained from micro-CT and histology, that the parasite moves with its anterior end toward the base of the Lieberkühn crypts, reaching deeper areas of the mucosa, and then returns to the apical part of the crypt, repeating this movement during intestinal colonization. However, we showed that this movement may have occurred at the *T*. *muris* L3 larval stage because it exhibited head-down behavior with an extroverted stylet at 17 d.p.i. on the intestinal surface (Fig 2). This would allow the first events of bacterial translocation, confirmed by the presence of bacteria in the submucosa at 17 d.p.i. and 22 d.p.i. (acute phase), not only in the chronic phase of infection at 35 d.p.i. (here), as previously shown [3].

This bacterial translocation is observed in trichuriasis and other parasitic infections, indicating the potential for secondary infection and inflammatory processes, which could exacerbate irritable bowel syndrome [34]. For example, *Escherichia coli*, a common bacterium in the intestinal microbiota, is associated with inflammatory bowel diseases such as Crohn’s disease and ulcerative colitis [28]. These findings suggest that trichuriasis and other intestinal parasites may be at risk of exacerbating chronic inflammatory bowel disease through bacterial translocation. The risk is associated with the fact that antiparasitic drugs are typically administered only after diagnosis, which often occurs during the chronic phase of infection, whereas bacterial translocation begins during the acute phase. Moreover, these drugs do not eliminate invasive bacteria.

Different parasitic infections can lead to an increase in the host’s body temperature (fever), typically during the acute phase of infection. This phenomenon is very common in malaria, frequently observed in schistosomiasis, and less common but still observed in soil-helminth transmitted infections [35–38]. The intestinal damage caused by the whipworm showed initial systemic signals coupled with an increase in body temperature at 22 d.p.i., corresponding to the acute phase, in which the L4 larval stage caused significant lesions, thus increasing bacterial translocation. During this same phase of infection, we observed increases in the Th1 cytokines TNF and IFN-γ, which are associated with lipopolysaccharide (LPS) induction, potentially interfering with the elevation of body temperature in the mice [39].

Chronic trichuriasis, whether associated with other parasitic infections or not, can result in anemia and weight loss in humans [2,40–42]. Similar observations have been reported in experimental infections with a high parasite load, including bacterial translocation and microbiota imbalance [3]. As part of the consequences of this inflammatory process, trichuriasis impact the gastrointestinal system, including increased peristalsis, which may lead to deficiencies in the absorption of micronutrients such as iron, zinc, and vitamin A [43,44]. In our results, we observed a reduction in fecal content in the large intestine of infected animals. This decrease may be associated with increased peristalsis, likely aimed at eliminating the parasite, and with the inflammatory response, which causes organ swelling and reduces the internal space [7]. Additionally, the observed increase in goblet cells and enterocytes stimulates the secretion of mucins, specifically Muc2 and Muc5 [50].

The digestive system associated with the intestinal microbiota, plays a critical role in nutrient metabolism and absorption [29,45] and has been identified as an important factor in mental health, particularly in conditions such as depression, as demonstrated in human infections with *Ascaris lumbricoides* [46]. These findings highlight the systemic impacts of the intestinal parasitic infections on nutrient absorption and dysbiosis, even in low-dose infections, which are more prevalent in endemic areas. The intestinal impacts and systemic consequences of the acute phase of trichuriasis were reflected in the changes observed in the peripheral blood, peritoneal cells, and spleen. Leukocytes in peripheral blood can migrate to different tissues via various stimuli, such as parasite antigens and microbial products, leading to either leukocytosis or leukopenia [47,48]. In C57BL/6 mice infected with a low-dose of *T*. *muris*, we observed specific changes in peripheral blood leukocyte counts. At 10 d.p.i., a significant decrease in the number of circulating monocytes was observed possibly due to migration to the cecum. By 22 d.p.i., there was a significant decrease in the number of lymphocytes and an increase in the number of basophils and neutrophils in the blood, indicating a response against L4 larvae and secondary infection by invasive bacteria. Similar to tissue mast cells, basophils release histamine and chemotactic factors that attract eosinophils and neutrophils, the latter play crucial roles in the initial phase of the immune response to infection, along with macrophages [48]. The increase in neutrophils on day 22 suggests that the presence of the parasite and increased bacterial translocation intensify and systemically activate the host immune response, which is reflected in peripheral blood cell counts.

Eosinophilia is common in helminth infections [49]; nevertheless, it is not consistently present in trichuriasis when analyzed in the bloodstream [2]. In infections with low parasitic loads, we observed an increase in eosinophils in the bloodstream from 10 d.p.i. onward. Other studies have described an increase in eosinophils in the intestinal mucosa during the chronic phase, which is regulated by the cytokine IL-5 and the chemokine CCL11 [3,50]. Our results revealed eosinophilia in the mucosa and submucosa of the infected intestine at all time points of infection (10, 17, 22 and 35 d.p.i.), indicating that this infection promotes the recruitment of eosinophils to the parasite niche even at the L2 larval stage.

The disruption caused by L2 larvae in the cecum triggers an inflammatory infiltrate composed of eosinophils, lymphocytes, neutrophils, and macrophages, leading to hyperplasia and hypertrophy of the mucosa and submucosa starting at 10 d.p.i. and intensifying the infection progresses. There is also an increase in enterocytes at 10 d.p.i. and goblet cells at 17 d.p.i., highlighting the role of enterocytes in the intestine’s absorption mechanism [51] and goblet cells in mucin secretion, which act as a chemical and mechanical barrier against intestinal pathogens [7,52]. IL-4 stimulates enterocytes to produce mucin, whereas IL-13 is involved in goblet cell hyperplasia [53,54]. We observed an increase in IL-4 in the cecum beginning at 10 d.p.i.. Furthermore, the hypertrophy of the muscular layer at 10 d.p.i. may be related to an increase in peristalsis activity as a strategy to eliminate the parasite. This increase in inflammatory infiltration, combined with hypertrophy of the muscular layer, partially explains how resistant strains resolve infection [55–57], whereas the cecum of susceptible mice becomes rigid and heavier in the chronic phase of infection [3].

Immunological signals resulting from infection start and finish in the cecum. Initially, there is a predominance of Th1/Th2 cytokines in the first days of the acute phase, which then transitions to the chronic phase characterized by a mixture of Th1/Th2/Th17 responses. Finally, in the chronic period, the adult worm of *T*. *muris* modulates the immune response at the site of insertion into the tissue, consolidating the mixed response. This is marked by an increase in the level of IL-10, a cytokine with potent anti-inflammatory properties that plays a central role in limiting the host immune response to pathogens. This mechanism helps prevent damage to the host and maintains normal tissue homeostasis [58,59]. This mixed cytokine profile and modulation of the immune response in the cecum have been previously observed in the chronic phase of trichuriasis with a high parasitic load [3]. These findings suggest that the immunomodulatory capacity of the excretion and secretion products of *T*. *muris* is associated with the complete formation of the bacillary band when it becomes an adult worm, influencing the response at the site of the parasite [3,60,61]. The modulation of the inflammatory response, as evidenced by the excretion and secretion products of *T*. *muris* [3], can confer neurological benefits to the host, given that helminth infections have been shown to prevent the cognitive dysfunction induced by early bacterial infections [62].

The damage related to the infection, including bacterial translocation, impacted the mesenteric lymph nodes, starting at 17 d.p.i., and expanded to the spleen and intraperitoneal cells [63]. During systemic infections, splenic macrophages play a significant role in eliminating bacterial infections, protecting against sepsis, and contributing to B and T cell activation in the white pulp [64]. Bacterial translocation begins in the acute phase when L3 larvae amplify intestinal tissue damage, persisting into the chronic phase [3]. From 17 d.p.i. to 35 d.p.i., we observed an increase in the white pulp of the spleen in infected mice, reflected in the increase in weight and size of this organ at 22 d.p.i. and 35 d.p.i.. These changes suggest that the lesions derived from the infection and bacterial translocation activated the lymphoid tissues of the spleen following sensitization of the mesenteric lymph nodes. Additionally, we observed an increase in the number of megakaryocytes in the red pulp in the chronic phase (35 d.p.i.), which are cells that assist in the coagulation process and tissue repair. This increase may be a consequence of the modulation of the immunological response as a result of excretory-secretory (ES) products of adult worms [64,65]. This finding is consistent with the increase in IL-10 levels observed in the cecum and mesenteric lymph nodes [58].

We observed an increase in IL-2 in the cecum at 17 d.p.i., period in which L4 favors bacterial translocation. These findings indicate that the presence of bacteria in the submucosa promotes T-cell activation and stimulates B-cell proliferation and differentiation. Additionally, the increase in IL-2 becomes evident later in the mesenteric lymph nodes, at 35 d.p.i., highlighting that the immune response at the site of infection occurs more rapidly than in the associated lymphoid tissues.

At 17 d.p.i., there was an increase in IL-6 and TNF production in peritoneal macrophages, which persisted until 22 d.p.i., along with an increase in IL-10 levels. At 35 d.p.i., increased IL-6 production was observed compared with that in the noninfected group, along with a notable decrease in TNF-α, suggesting that IL-6 had a stronger effect on the Th1 response profile in noninfected individuals. The number of intraperitoneal cells, mainly monocytes and neutrophils recruited during infection, was significantly greater in the infected group than in the noninfected group, suggesting peritonitis, as expected and as previously shown in high-dose experimental trichuriasis in the chronic phase [3]. These cells work to prevent systemic inflammation, recruiting other cells and molecules involved in inflammatory or repair mechanisms, depending on the cytokine profile [66–68]. The differentiated macrophages isolated from infected mice were more responsive to LPS treatment than those isolated from uninfected mice were, indicating an activated state [3,69]. Nitric oxide is associated with bacterial and cytotoxic reactions, highlighting the important role of these cells in combating bacterial translocation during trichuriasis [70].

Based on our findings of bacterial translocation in the chronic phase, and the location and systemic changes observed in the acute phase, and the ultrastructural evidence, and the cellular and immunological alterations, resulted in a complementary question as follows: At what point does bacterial translocation occur in acute experimental trichuriasis? For this purpose, we used fluorescence *in situ* hybridization (FISH) analyses.

Our data complement previous observations in the chronic phase of high parasite load infection [3] and enhance this translocation mapping. The limitation of using low-dose infections in C57Bl/6 mice was the difficulty in detecting sufficient quantities of bacteria to demonstrate in FISH results. Therefore, we used the high-dose infection model with Swiss mice for the FISH and SEM experiments. We show that bacteria invade during the acute phase, particularly when tissue lesions are exacerbated by L3 larvae (17 days) and L4 larvae (22 days). These results suggest that bacterial translocation is a gradual process involving damage caused by the nematode and the mechanical action of the parasite’s attachment and movement. This process leads to increased and cumulative internalization of microorganisms into deeper layers of the mucosa and submucosa. [71] reported that the effectiveness of drug treatment is very high for *A*. *lumbricoides* infections, whereas cure rates for *T*. *trichiura* infections are low. This difference in efficacy may be due to the intratissue behavior of the parasite and the possible detoxification activity of anthelminthic compounds by the bacillary band, increasing the resistance of *Trichuris* to conventional doses of benzimidazoles, which are normally efficient for other nematodes [16,61]. The dynamics and synergism between whipworms and bacteria in the infection and inflammatory process could open new therapeutic strategies, combining anthelmintics with antibiotics, for treating parasites that use intestinal tissue as a parasitic niche, causing bacterial translocation.

By integrating our results with recent advances in understanding the intratissue behavior of *Trichuris* nematodes [13,33], we explored and correlated different techniques, highlighting advanced microscopy methods such as SEM, lightsheet microscopy and micro-CT. This approach enabled us to summarize the mechanisms of attachment and movement of the parasite within the tissue, particularly bacterial translocation (S1 Movie). The 3D printed model created in this study has the potential to improve scientific insights into the host–parasite relationship, offering a multidimensional perspective that can improve teaching in higher education. This model can also support the training of professionals in parasitology by providing an opportunity to associate image observation with tactile experiences. Additionally, these printed biological and medical models can be valuable tools in the learning process for students, including those with visual impairments [72–75].

**Fig 16:**
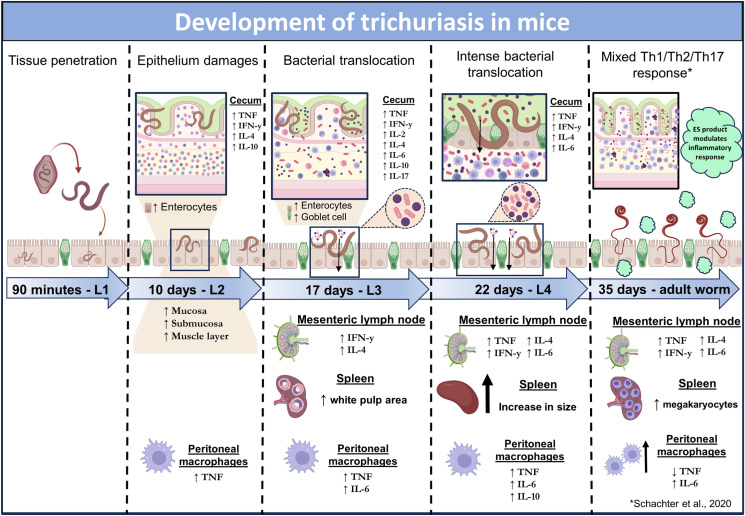
Dynamics of infection development. Scheme showing the intricate interplay among host immune responses, the timing of bacterial translocation in the gut, changes in the intestinal layers, spleen architecture, peritoneal macrophages, and parasite survival strategies during the development of *Trichuris muris* infection. Created with BioRender.com and then modified.

In conclusion, our study reveals a cascade of events triggered by the parasite, affecting not only the intestine and cecum but also the induction of systemic immunological changes detectable in the peripheral blood, peritoneum, and lymphoid organs as early as the L2 larval stage. At 10 d.p.i. (days post-infection), L2 larvae had already initiated intestinal mucosal damage, as evidenced by mucosal and submucosal thickening due to inflammatory infiltrates. Notably, bacterial infiltration into the intestinal submucosa was observed only at 17 d.p.i., which coincided with a balanced immune response shift toward the cecum initially and later toward the mesenteric lymph nodes (22 d.p.i. and 35 d.p.i.), revealing the immunomodulatory potential of *T*. *muris* ES products at the site of the parasite. Peritoneal macrophages play a crucial role in the host defense against bacterial invasion, increasing IL-6 and TNF production as bacterial infiltration deepens. In the chronic phase, these macrophages become more numerous and activated, possibly producing regulatory cytokines to mitigate damage while combating infection. The impact of trichuriasis is evident early in the L2 stage. However, it intensifies as the parasite matures (L3 and L4 stages) into adult worms, and bacterial translocation increases. Even in the acute phase, the spleen undergoes changes, indicative of the host’s systemic response. L4 larvae possess immunomodulatory capabilities, initially affecting the cecum and later the mesenteric lymph nodes. Our findings underscore the intricate interplay between host immune responses, the gut microbiome, and parasite survival strategies, showing that the process of bacterial translocation is not exclusively due to mucosal tissue rupture but is instead facilitated by the mechanical action of nematodes moving within the tissue, promoting the invasion of microorganisms. This is crucial for understanding clinical outcomes and devising new therapeutic approaches (Fig 16).

## Supporting information

S1 Fig Body temperature. The body temperature of the animals was measured using an infrared thermometer, which revealed an increase in body temperature on the 22nd day of infection by *Trichuris muris*. The significance of differences between the noninfected and infected groups (n = 6) was determined via Student’s t test, and asterisks indicate statistically significant differences. * p value ≤ 0.05.
(TIF)

S2 Fig MCP-1 Peritoneal macrophage. Graph representing the levels of monocyte chemoattractant protein-1 (MCP-1), a chemokine involved in macrophage recruitment and commonly associated with inflammation, throughout the course of *Trichuris muris* infection. No significant differences were observed between the noninfected and infected groups. * p value > 0.05. ** Not identified.
(TIF)

S3 Fig Bacteria on the mucosal surface that adhere to the nematode cuticle. SEM of the fractured cecum of a mouse after 17 days of infection, where it is possible to visualize the goblet cells (Gc) and the *Trichuris muris* (asterisk) inserted in the intestinal mucosa, causing rupture (arrowhead) in the mucosa, the region inside the syncytial tunnel (tu), and bacteria (arrows) on the mucosal surface that adhere to the cuticle of the parasite. Scale bar represents 10 µm.
(TIF)

S4 Fig Photographs showing different sides of the 3D printed models. (A) Photograph of the printed model being held in the palm of the hand, showing the penetrating parasite. Scale bars represent 3 cm. (B) Photograph of the printed model being held in the palm of the hand, showing the posterior region of the parasite on the mucosal surface. Scale bars represent 3 cm. (C) Photograph of the printed model on the support with identification, showing the parasite inserted into and breaking through the intestinal mucosa. Scale bars represent 3 cm. (D) Photograph of the printed model on the support with identification, showing the posterior region of the parasite on the mucosal surface. Scale bars represent 3 cm.
(TIF)

S5 Fig Development of trichuriasis in mice. Scheme showing the tissue damage caused by the nematode, bacterial translocation in the gut, and the immunomodulation by ES products. Created with BioRender.com and then modified.
(TIF)

S1 Table Prevalence of cells observed in peripheral blood smears. Significance between the noninfected and infected groups (n = 9) was determined via Student’s t test, and asterisks indicate statistically significant differences. * p value ≤ 0.05. ** Not identified.
(DOCX)

S2 Table Body weight of C57BL/6 mice measured in grams. The sample size (“N”) included 5 control animals and 9 infected animals, except on day 22 (13 infected) and day 35 (16 infected). Statistical analysis was performed using Student’s t-test, and no significant differences were observed.
(DOCX)

S1 Movie Animation showing how nematode movement promotes bacterial translocation. Animation showing the intestine (pink), the parasite (yellow) and bacteria on the surface (inset: purple and green), suggesting how nematode attachment promotes bacterial translocation and secondary infections.
(MP4)
