# Supplementary figures and images for: Experimental trichuriasis: Changes in the immune response and bacterial translocation during acute phase development illustrated with 3D model animation
Source: PLoS Negl Trop Dis. 2025 Feb 3;19(2):e0012841. doi: 10.1371/journal.pntd.0012841 (PMC11805410; doi:10.1371/journal.pntd.0012841)

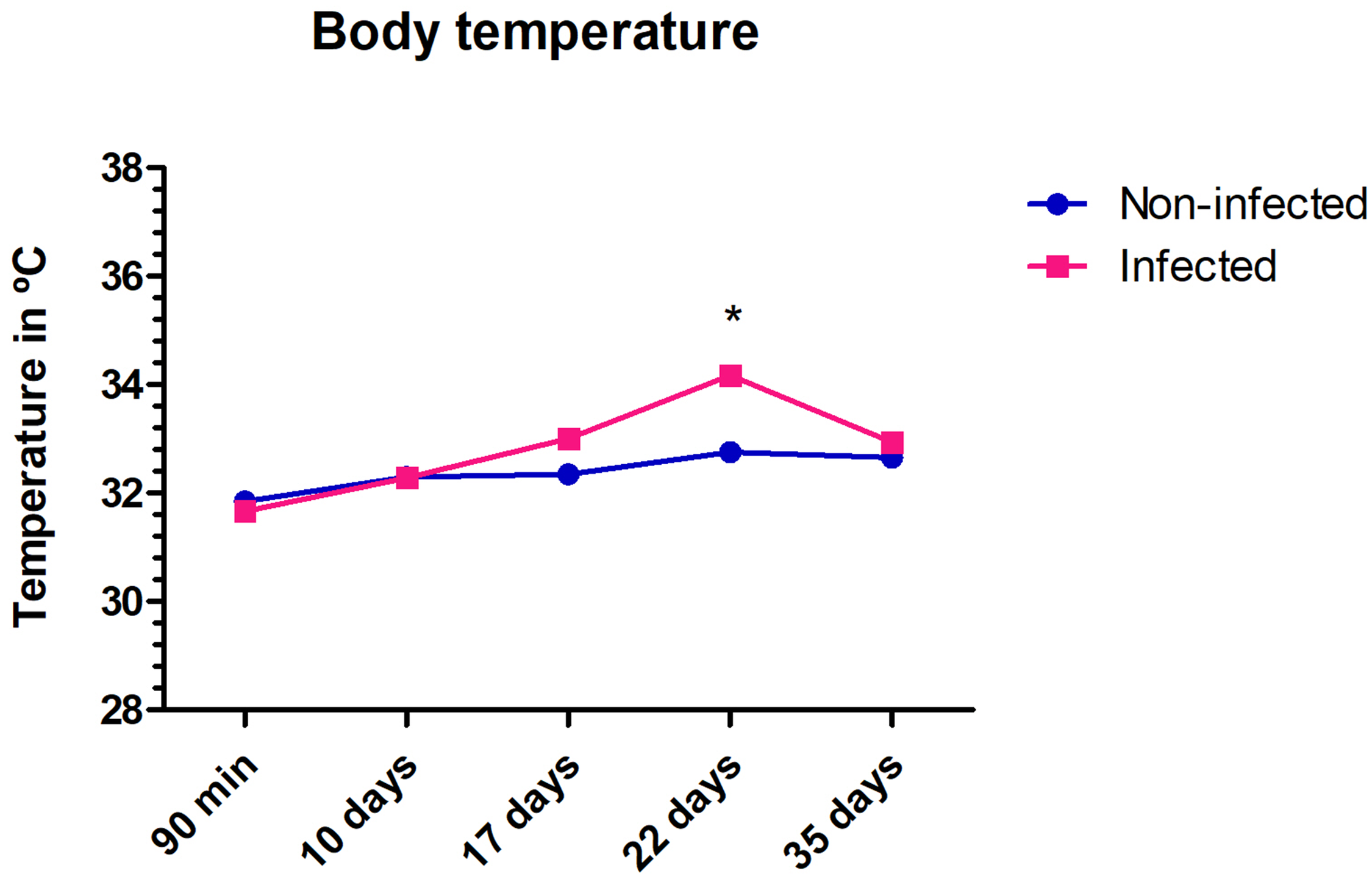

Supplement: S1 Fig — The body temperature of the animals was measured using an infrared thermometer, which revealed an increase in body temperature on the 22nd day of infection by Trichuris muris. The significance of differences between the noninfected and infected groups (n = 6) was determined via Student’s t test, and asterisks indicate statistically significant differences. * p value ≤ 0.05. (TIF) [file pntd.0012841.s001.tif]

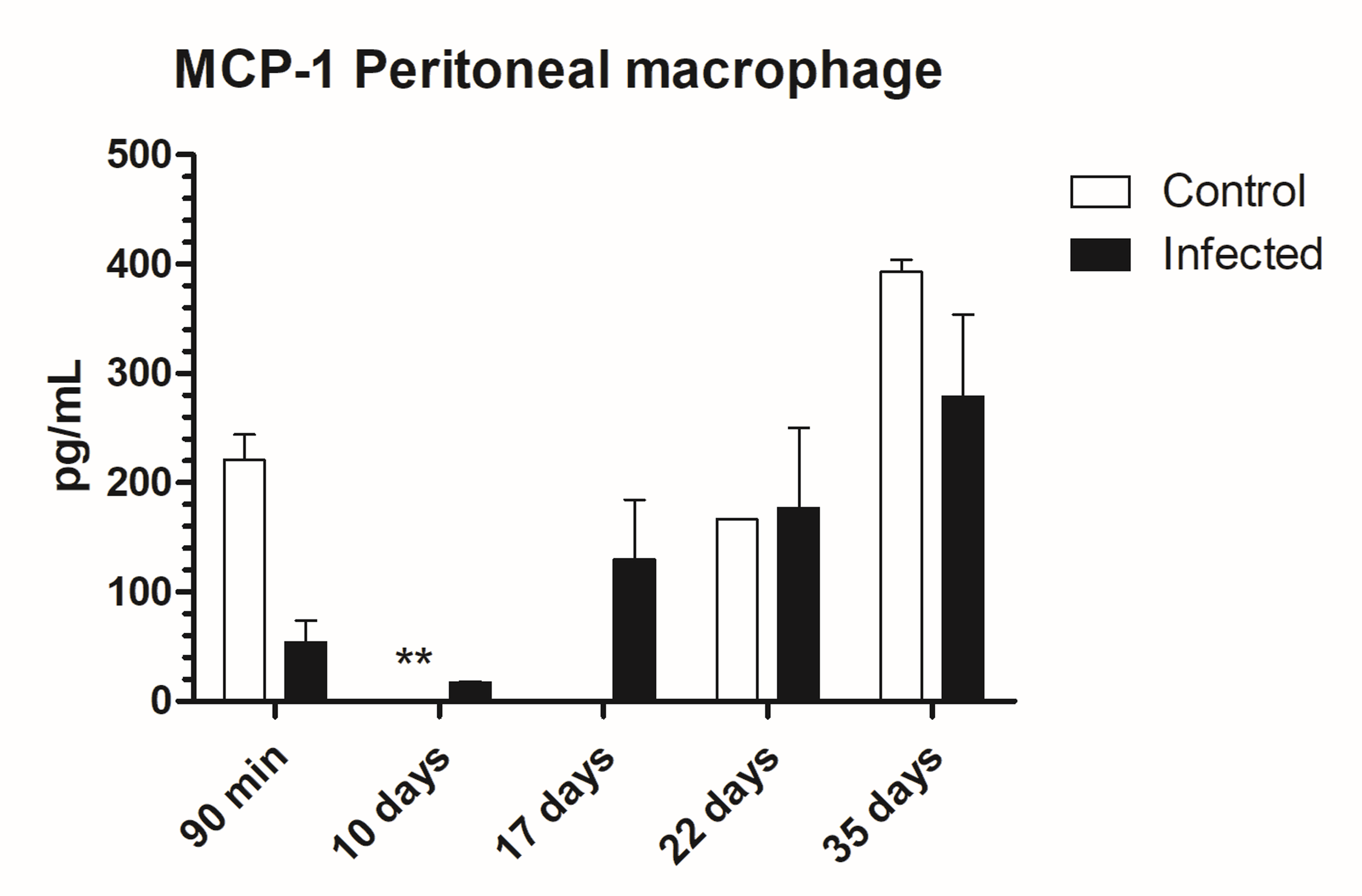

Supplement: S2 Fig — Graph representing the levels of monocyte chemoattractant protein-1 (MCP-1), a chemokine involved in macrophage recruitment and commonly associated with inflammation, throughout the course of Trichuris muris infection. No significant differences were observed between the noninfected and infected groups. * p value > 0.05. ** Not identified. (TIF) [file pntd.0012841.s002.tif]

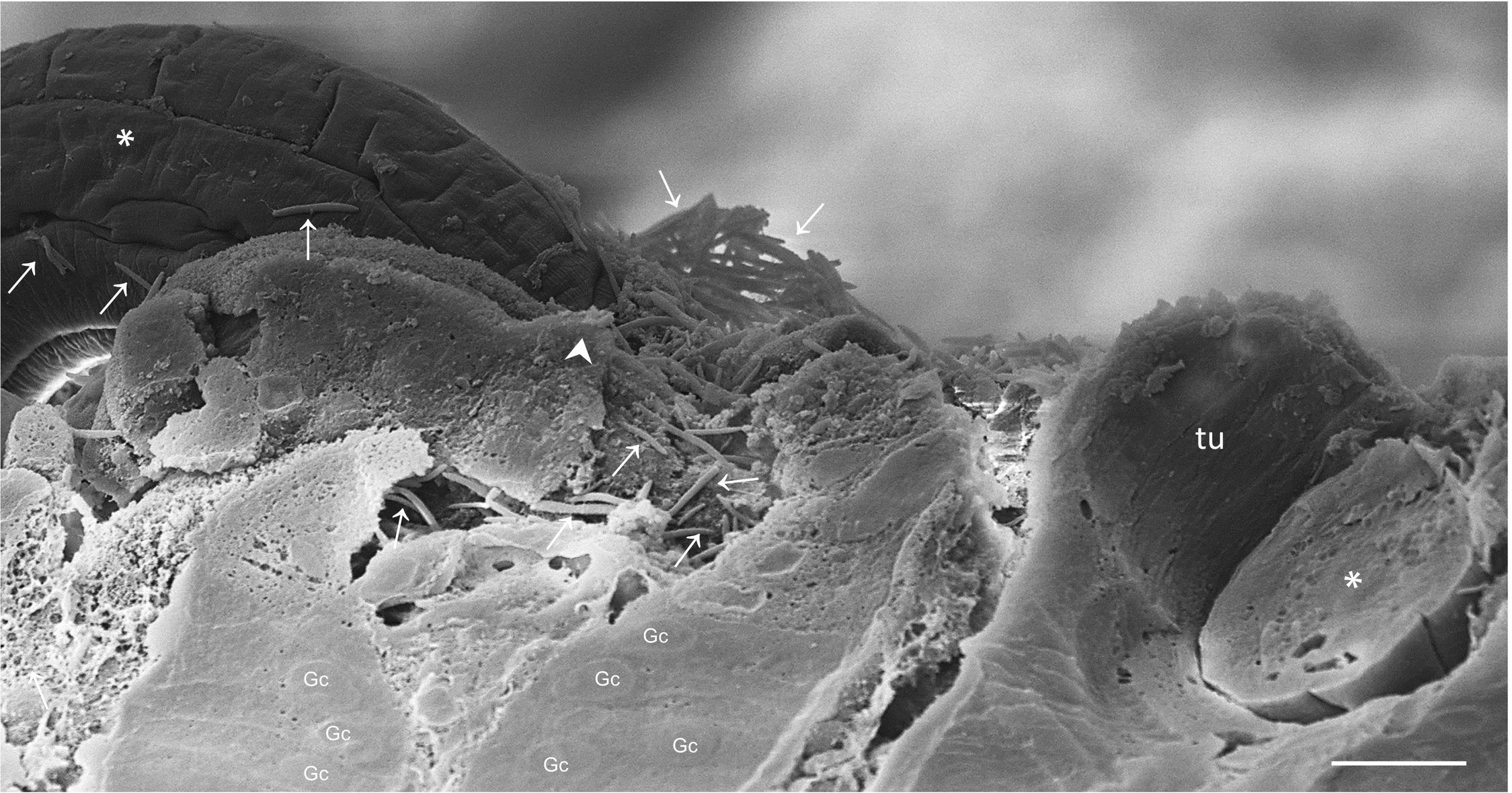

Supplement: S3 Fig — SEM of the fractured cecum of a mouse after 17 days of infection, where it is possible to visualize the goblet cells (Gc) and the Trichuris muris (asterisk) inserted in the intestinal mucosa, causing rupture (arrowhead) in the mucosa, the region inside the syncytial tunnel (tu), and bacteria (arrows) on the mucosal surface that adhere to the cuticle of the parasite. Scale bar represents 10 µm. (TIF) [file pntd.0012841.s003.tif]

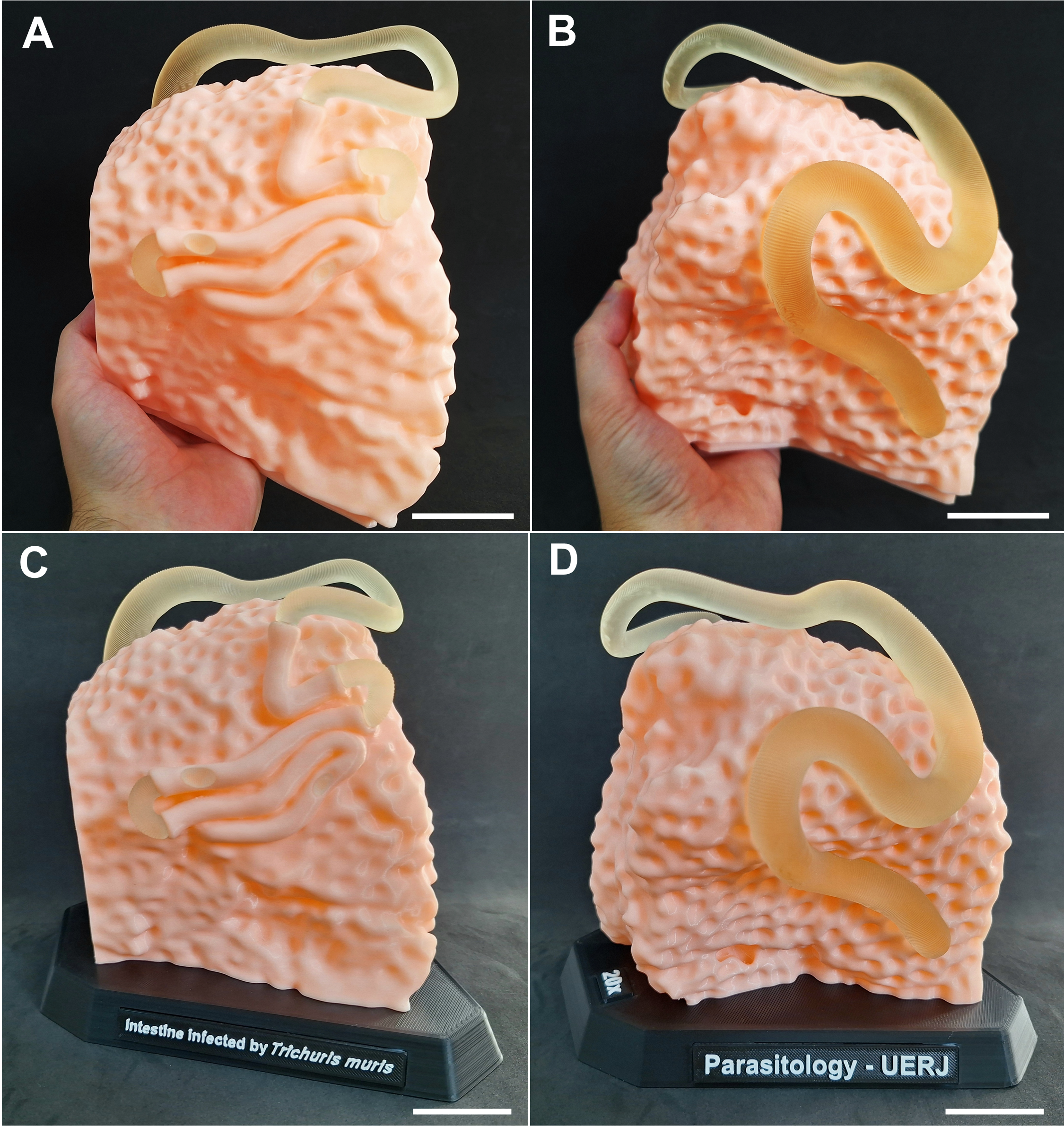

Supplement: S4 Fig — (A) Photograph of the printed model being held in the palm of the hand, showing the penetrating parasite. Scale bars represent 3 cm. (B) Photograph of the printed model being held in the palm of the hand, showing the posterior region of the parasite on the mucosal surface. Scale bars represent 3 cm. (C) Photograph of the printed model on the support with identification, showing the parasite inserted into and breaking through the intestinal mucosa. Scale bars represent 3 cm. (D) Photograph of the printed model on the support with identification, showing the posterior region of the parasite on the mucosal surface. Scale bars represent 3 cm. (TIF) [file pntd.0012841.s004.tif]

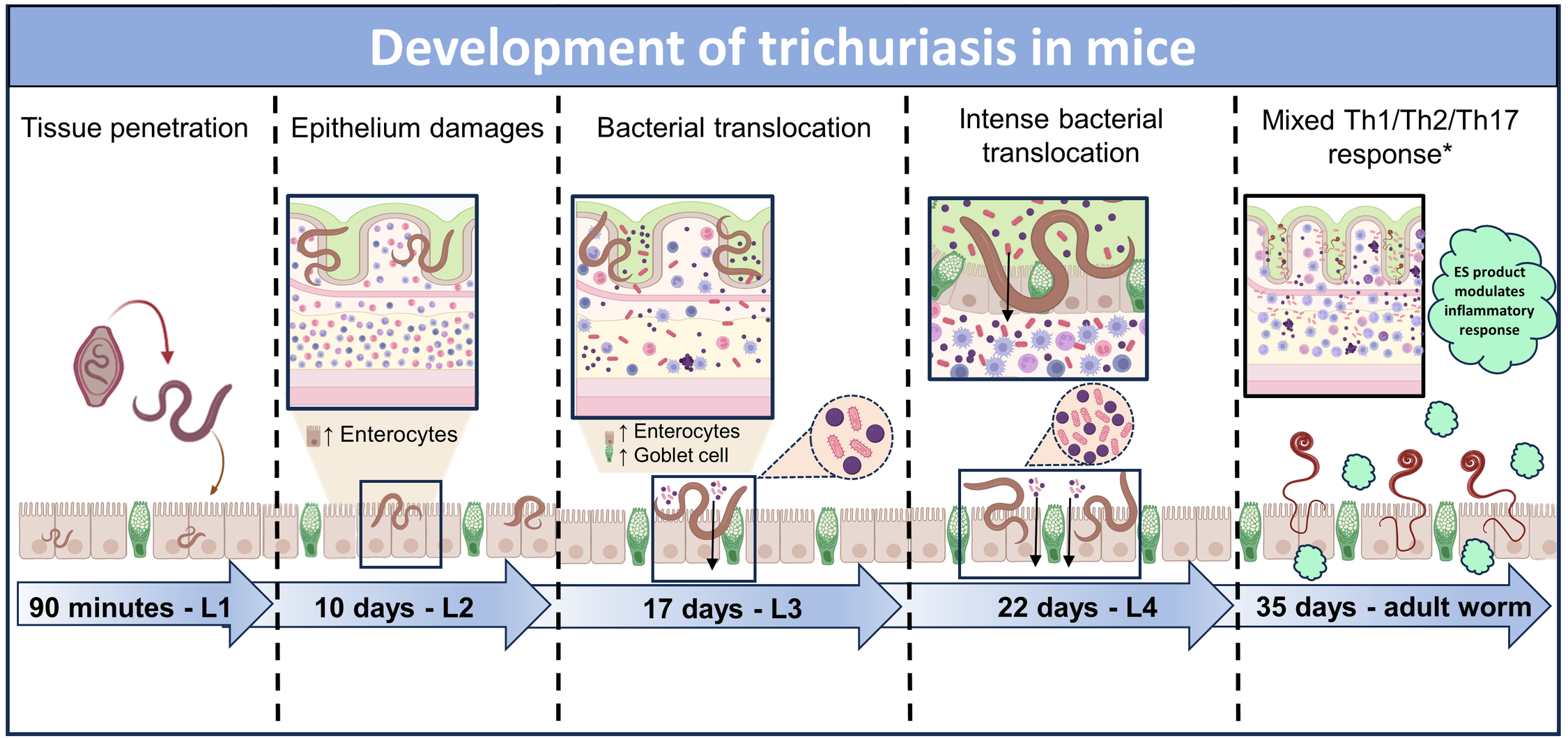

Supplement: S5 Fig — Scheme showing the tissue damage caused by the nematode, bacterial translocation in the gut, and the immunomodulation by ES products. Created with BioRender.com and then modified. (TIF) [file pntd.0012841.s005.tif]
