# Supplementary material for: Experimental trichuriasis: Changes in the immune response and bacterial translocation during acute phase development illustrated with 3D model animation
Source: PLoS Negl Trop Dis. 2025 Feb 3;19(2):e0012841. doi: 10.1371/journal.pntd.0012841 (PMC11805410; doi:10.1371/journal.pntd.0012841)
Supplement: S1 Table — Significance between the noninfected and infected groups (n = 9) was determined via Student’s t test, and asterisks indicate statistically significant differences. * p value ≤ 0.05. ** Not identified. (DOCX) [file pntd.0012841.s006.docx]

| **S1 –** **Prevalence of cells observed in peripheral blood smears** | | | | | |  |  |  |
| --- | --- | --- | --- | --- | --- | --- | --- | --- |
|  |  | **Lymphocyte** (%)  (Mean±ErroPad) | **Neutrophil** (%)  (Mean±ErroPad) | **Monocyte** (%)  (Mean±ErroPad) | **Eosinophil** (%)  (Mean±ErroPad) | | **Basophil** (%)  (Mean±ErroPad) | **Other cells** (%)  (Mean±ErroPad) |
| **90 min** | Noninfected | 49.500 ± 8.500 | 27.000 ± 8.000 | 21.500 ± 0.5000 | ** | | 1.500 ± 0.5000 | ** |
|  | infected | 55.000 ± 4.239 | 23.444 ± 3.786 | 15.111 ± 1.859 | ** | | 3.000 ± 0.8864 | ** |
|  | p value | p = 0.5914 | p = 0.6981 | p = 0.1549 | ** | | p = 0.4444 | ** |
| **10 days** | Noninfected | 46.200 ± 4.641 | 17.200 ± 2.375 | 31.800 ± 6.288 | 1.400 ± 0.2449 | | 1.800 ± 0.2000 | ** |
|  | infected | 53.667 ± 5.783 | 21.556 ± 5.164 | 19.222 ± 2.235 | 4.333 ± 0.8819 | | 1.333 ± 0.2108 | ** |
|  | p value | p = 0.4012 | p = 0.5597 | p = 0.0400* | p = 0.0330* | | p = 0.1479 | ** |
| **17 days** | Noninfected | 41.500 ± 0.5000 | 23.000 ± 12.000 | 26.500 ± 10.500 | 2.500 ± 0.2887 | | 3.500 ± 0.5000 | ** |
|  | infected | 48.000 ± 3.606 | 13.000 ± 1.414 | 28.000 ± 3.564 | 6.200 ± 0.4899 | | 4.600 ± 1.030 | ** |
|  | p value | p = 0.3309 | p = 0.2000 | p = 0.8612 | p = 0.0007* | | p = 0.5557 | ** |
| **22 days** | Noninfected | 60.375 ± 3.669 | 12.750 ± 1.556 | 21.750 ± 3.712 | 2.250 ± 0.4532 | | 2.000 ± 0.3273 | ** |
|  | infected | 44.538 ± 3.777 | 19.308 ± 1.461 | 27.231 ± 3.373 | 4.846 ± 0.5044 | | 3.462 ± 0.3859 | ** |
|  | p value | p = 0.0111* | p = 0.0084* | p = 0.3052 | p = 0.0023* | | p = 0.0168* | ** |
| **35 days** | Noninfected | 66.750 ± 2.287 | 17.250 ± 3.728 | 14.500 ± 1.041 | 2.333 ± 0.5578 | | ** | ** |
|  | infected | 60.188 ± 2.626 | 14.000 ± 1.656 | 19.750 ± 1.647 | 4.867 ± 0.5243 | | ** | ** |
|  | p value | p = 0.2450 | p = 0.4017 | p = 0.1394 | p = 0.0116* | | ** | ** |
