# Supplementary material for: Experimental trichuriasis: Changes in the immune response and bacterial translocation during acute phase development illustrated with 3D model animation
Source: PLoS Negl Trop Dis. 2025 Feb 3;19(2):e0012841. doi: 10.1371/journal.pntd.0012841 (PMC11805410; doi:10.1371/journal.pntd.0012841)
Supplement: S2 Table — The sample size (“N”) included 5 control animals and 9 infected animals, except on day 22 (13 infected) and day 35 (16 infected). Statistical analysis was performed using Student’s t-test, and no significant differences were observed. (DOCX) [file pntd.0012841.s007.docx]

| **Infection time** | **Noninfected**  **(Mean±ErroPad)** | **Infected**  **(Mean±ErroPad)** | **p value** |
| --- | --- | --- | --- |
| 90 min | 18.49 ± 1.90 | 18.43 ± 1.69 | p = 0.8981 |
| 10 days | 20.20 ± 2.78 | 20.10 ± 1.48 | p = 0.6993 |
| 17 days | 22.01 ± 1.89 | 21.01 ± 1.50 | p = 0.3543 |
| 22 days | 23.07 ± 2.45 | 22.37 ± 3.40 | p = 0.7750 |
| 35 days | 24.34 ± 1.74 | 24.63 ± 1.73 | p = 0.8417 |

**S2 – Body Weight of C57BL/6 Mice (grams)**
